# Supplementary material for: Characterization of a Novel Phage ΦAb1656-2 and Its Endolysin with Higher Antimicrobial Activity against Multidrug-Resistant Acinetobacter baumannii
Source: Viruses. 2021 Sep 16;13(9):1848. doi: 10.3390/v13091848 (PMC8473069; doi:10.3390/v13091848)
Supplement: Supplementary file 1 [file viruses-13-01848-s001.zip › viruses-1346213-supplementary.pdf]

Supplementary Figure 1.

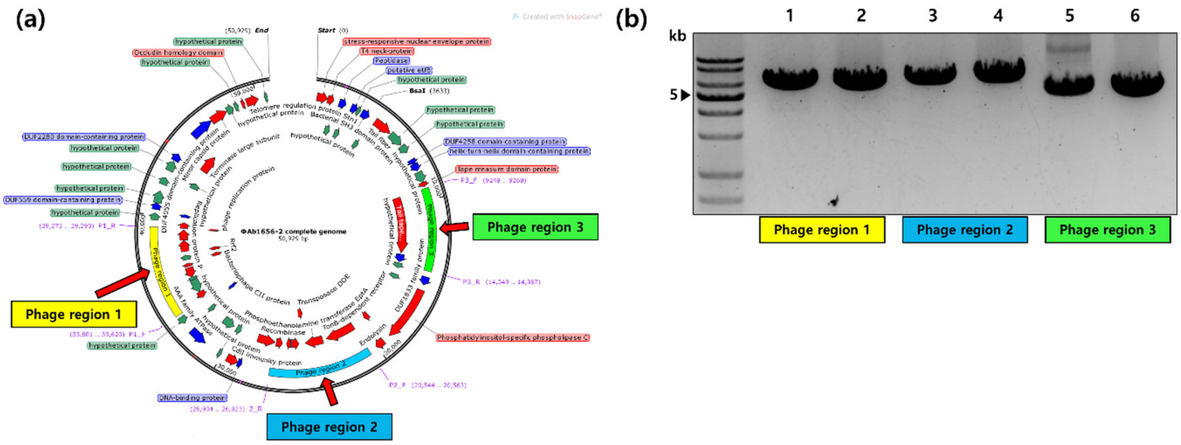

Figure S1. Identification of ΦAb1656-2 genome from ΦAb1656-2 and *A. baumannii* 1656-2 by PCR. (a) Location of phage regions in the whole phage sequence. (b) The phage regions was amplified using *A. baumannii* 1656-2 chromosomal DNA as templates in lane 1, 3, 5 and ΦAb1656-2 genomic DNA as templates in lane 2, 4, 6. The PCR amplicon size of phage regions are 6.4 kb, 6.4 kb and 5.1 kb, respectively.

Supplementary Figure 2.

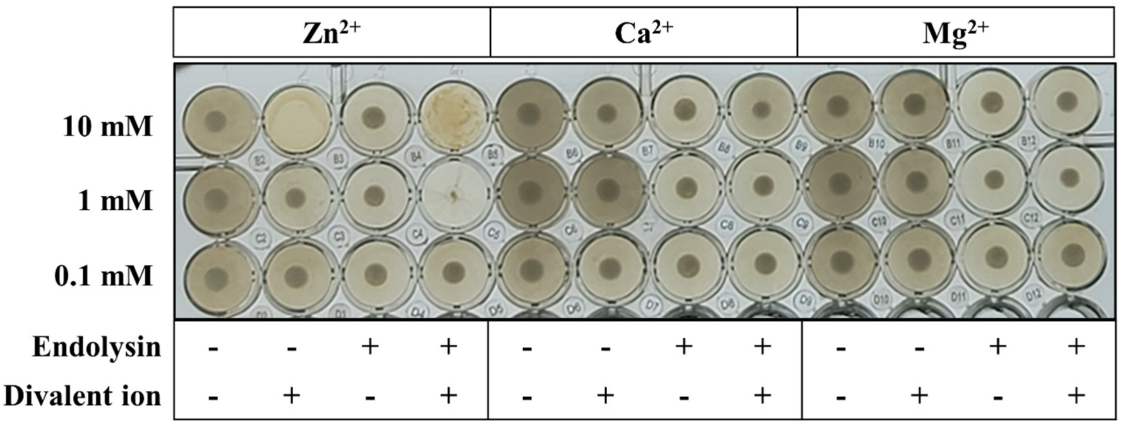

Figure S2. Effects of divalent cation ions on the antibacterial activity of AbEndolysin. The concentration of AbEndolysin was fixed, and the divalent ions Zn^{2+}, Ca^{2+}, and Mg^{2+} were used at concentrations of 0.1 mM, 1 mM, and 10 mM, respectively. Total volume of each well was fixed as 200 µl with fresh MHB.

Supplementary Figure 3.

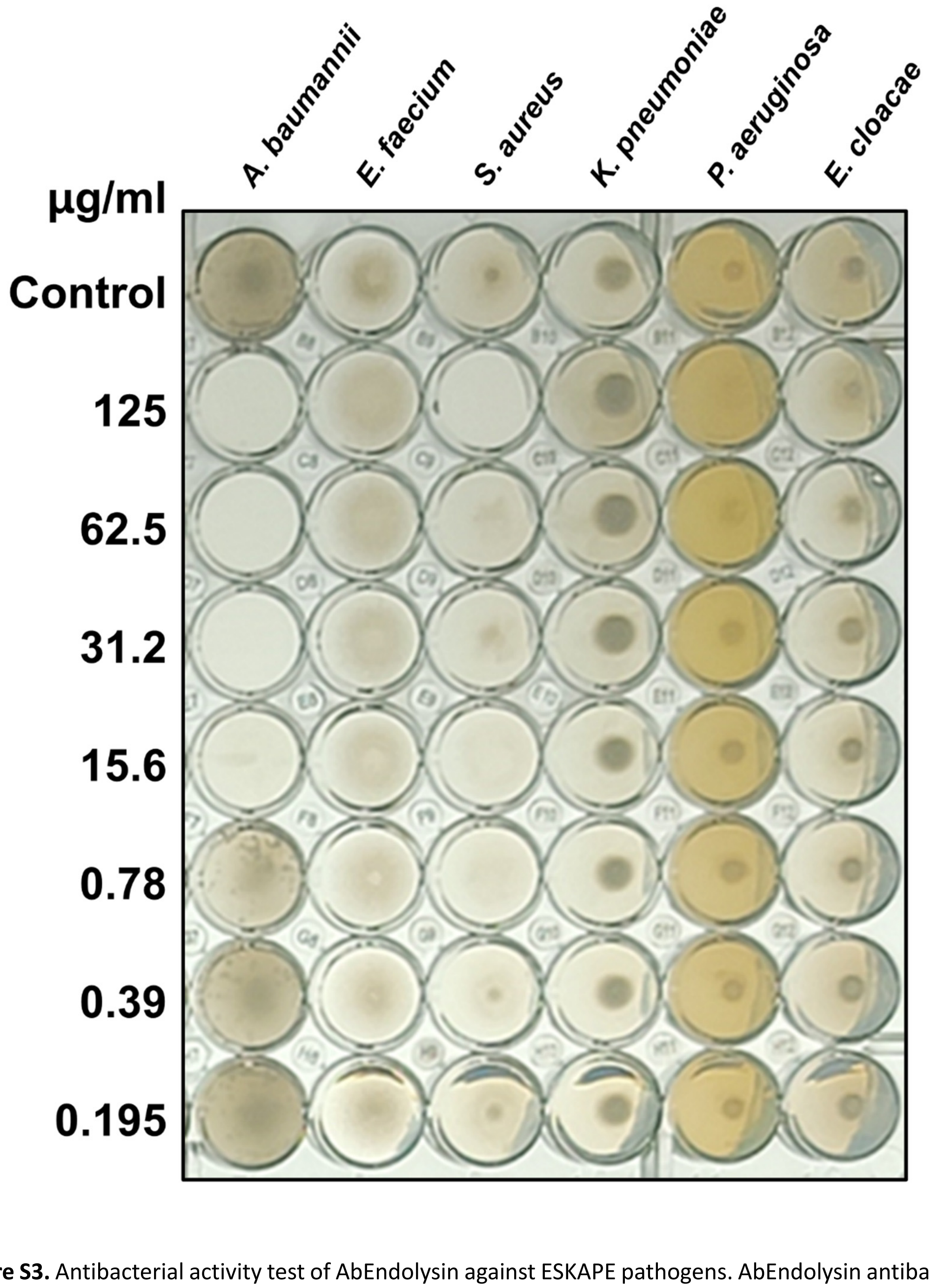

Figure S3. Antibacterial activity test of AbEndolysin against ESKAPE pathogens. AbEndolysin antibacterial activity against ESKAPE pathogens (*Enterococcus faecium* (clinical strain), *Staphylococcus aureus* ATCC 33591, *Klebsiella pneumoniae* ATCC 13883, *A. baumannii* ATCC 17978, *Pseudomons aeruginosa* ATCC 27853 and *Enterobacter cloacae* (clinical strain)). AbEndolysin was serially diluted two-fold from the highest concentration of 125 µg/ml to confirm its inhibitory concentration. Fresh bacterial culture in MHB was used as control.

Supplementary Figure 4.

(a)

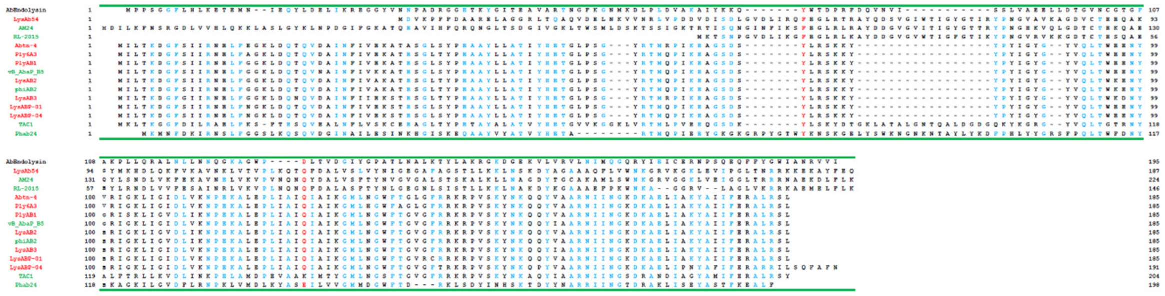

>YP_009882155.1 LysAb54
>APD20282.1 endolysin [Acinetobacter phage AM24]
>AJG41873.1 endolysin [Acinetobacter phage RL-2015]
>YP_009624620.1 endolysin [Acinetobacter phage vB_AbaP_D2]
>YP_009190475.1 putative endolysin [Acinetobacter phage vB_AbaP_PD-6A3]
>YP_008058242.1 EF hand domain protein [Acinetobacter phage Abp1]
>YP_009610435.1 endolysin [Acinetobacter phage vB_AbaP_B5]
>HM755898.1 Acinetobacter phage phiAB2 endolysin (lysAB2) gene
>ADX62345.1 endolysin [Acinetobacter phage phiAB2]
>YP_008060133.1 putative endolysin [Acinetobacter phage AB3]
>KF548002.1 Acinetobacter phage ABP-01 putative endolysin (lysABP-01) gene
>KF548003.1 Acinetobacter phage ABP-04 putative endolysin (lysABP-04) gene
>AZF88457.1 endolysin [Acinetobacter phage TAC1]
>QXM18610.1 endolysin [Acinetobacter phage Phab24]

(b)

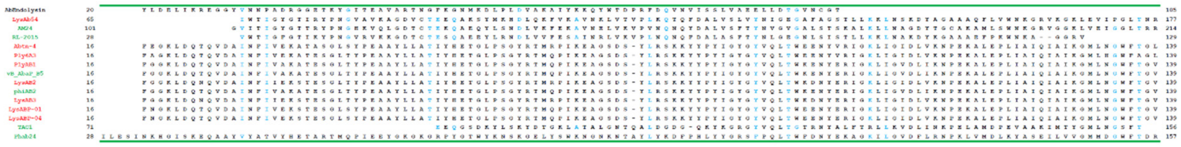

>YP_009882155.1 LysAb54
>APD20282.1 endolysin [Acinetobacter phage AM24]
>AJG41873.1 endolysin [Acinetobacter phage RL-2015]
>YP_009624620.1 endolysin [Acinetobacter phage vB_AbaP_B5]
>YP_009190475.1 putative endolysin [Acinetobacter phage vB_AbaP_PD-6A3]
>YP_008058242.1 EF hand domain protein [Acinetobacter phage Abp1]
>YP_009610435.1 endolysin [Acinetobacter phage vB_AbaP_B5]
>HM755898.1 Acinetobacter phage phiAB2 endolysin (lysAB2) gene
>ADX62345.1 endolysin [Acinetobacter phage phiAB2]
>YP_008060133.1 putative endolysin [Acinetobacter phage AB3]
>KF548002.1 Acinetobacter phage ABP-01 putative endolysin (lysABP-01) gene
>KF548003.1 Acinetobacter phage ABP-04 putative endolysin (lysABP-04) gene
>AZF88457.1 endolysin [Acinetobacter phage TAC1]
>QXM18610.1 endolysin [Acinetobacter phage Phab24]

Figure S4. Multiple sequence alignment of AbEndolysin with another *Acinetobacter* phage derived endolysin. Thirteen *Acinetobacter* phage derived endolysins amino acid sequence was compared. Red letters (Abtn-4, ply6A3, plyAB1, LysAB2, LysAB3, LysABP-01 and LysABP-04) indicate reported endolysins and green letters (vB_AbP_B5, phiAB2, TAC1, Phab24, AM24 and RL-2015) indicate endolysins sequence obtained from the NCBI protein database.

Table S1: list for the genome predicted 68 putative ORFs

Protein_ID	Start	End	Strand	Product	Protein Seq
phageDNA_00001	192	857	+	stress-responsive nuclear envelope protein	MKIIYTRIAAAAALETGIIANPDYYENPNLKAKEVIIYGNYPKIQKDYESLEVPVEVRKLEVPQKTTLATVNVAVGITPELQAVMDDAKAECEKVVEENTQLKQKIAILEQAGGNQSELLSENSRLKDAAVLADKALKDAEAQVVGIKTEFEAFKNDIPAMQARIAELEAGKAAENPATETAANDFENWSNDQLKEYLASKNIGYKPSATKAELLKLIPKE*
phageDNA_00002	862	1251	+	T4_neck-protein	MSFITVDDANSILGSDFAPDSDKARLVKLANVWMKNRIGFVPDPIDPLLKDAACEIIKGILAKVIYNGKEQQLKRKKVKADSVESEKEFQDGSEAISSFEQIAIDFIDSLDLKDPNASFNGFGIPLYRA*
phageDNA_00003	1208	1621	+	hypothetical protein Peptidase	MQVLMALAYHFTGHDMGLRDEIQADIAEAFNDDLADAIHTFTCERISKTNWDPKTETYVEVKENYSGRGVLFGSYSQYEIQTLGVLATDKKATVLQNEVTMTPKIEDEWLTALGSFRVINIQQDPANTIWKCQLRKV*
phageDNA_00004	1600	2034	+	hypothetical protein	MSASKSVGAKMVNPDYVPEWYISPFQHVQYTLARNQLHMDLLFEDMDKADQFLDMGADAQVSTFSDGAYAIVQIGDTADKDRIQVYGLLLHEAVHVWQKIKKLMGEREPSSEFEAYSIQAIAQDLFKMYEESEVNDGMEGEKAN*
phageDNA_00005	2006	2374	+	hypothetical protein	MGWKGKKPTEFSFDVSKAAEDQVKHIVMDTVQSLVNLSPVDTGAYRASHIVSVGSADFGVREPETNPINDAAIQAMKIKLGNLVYIQNNKAYGPRLENGWSDQAPQGIYGLTFNFISQKYGG*
phageDNA_00006	2382	2774	+	putative Electron transfer flavoprotein subunit beta (etfB)	MTLEQTRQAIIDRMQSFTGIAQERIQYPNAPDFTVPTKGVWCRLTIAGGPSFTSGIADKPCTRRTGNIMIQCFDRLHTGEKAVTVLSDALLAHFEYFTIEHLECLNGQSIYAGKDADFIQYNVSIGYKVN*
phageDNA_00007	2776	2994	+	hypothetical protein	MSCMLTLEEIEIKRQELERHLEDVMSVELSKWQSENKLCVSDVNIRLANVVSLGGPKHNVVTGVSVDLDNEL*
phageDNA_00008	3103	3624	+	bacterial SH3 domain protein	MKKSTLGWGAAGLVALGIFGSGNDNSPKQTSDSENAQSAVEEVIESKYINTNSLNIRDKPNGQVVGKLGRGEKVDIYEMKGNWARISLNSSSPQWLSTKLLCETDGCFKQKSRSTTSNNYQALKSHPHHSERKQKKTYYDSDCSCAVVDYCVGPRGGHYCITSGGNKRYKPRY*
phageDNA_00009	3721	4074	+	hypothetical protein	MSNFVFKRGDTFNLNLQLVDMDEALQYPPDDVRRAIDLTGYTFTSQVKALADGATVATLTCTALSQSTQKGWLNIKSSASTATWPLGLCQMDIKAVVSGTTQHTETLTFQVIDGVTA*
phageDNA_00010	4074	5252	+	putative tail fiber	MANLVFKFSWDHRPFPYNASQGKRQFMLPFASGIPNLAPNFSQVVGTAAISQGGTGATTAAGARANLGAAASGVNSDISELKGLTTPLSISQGGLGADNAQTARMNLGLGTAAVLASTTSQYDPTPGRALRVGDWGIGAEGSRVSDMVAPLNNGFFRTDDTLTNDTGNSIGPYGFFLHCTRRSMGLYTNGSHSFQLGKAASYSALKYRFNNSGTWSNWFNLLTAQNTTTDGNGFIKAASPVVKLFQSHIELNNDAAKQPITFDKLGTGDYLIKGSLGFAQEGWYIEVPKDANGNTVVAVEYSTLENGDLSIKTYKRKFDVEKAAIVADLENPLDIPEGRWIDIRLHEEPEPEPEEPLSETPVDFQPTNLSQAVAAAMNGVEPPEISETDETL*
phageDNA_00011	5305	6222	+	hypothetical protein	MSSGAKIRLYACEEAVLGTTPANPVWYTVRRVTDSLTENVTTEDSSEVVDSRFRQGAVVTEAEVTGQLEFELSLGTFDLFLNVLAFNNWAANALSFGGGVRKSLTLVKVFEDIGQVFIYRGIQVNTGEMTIQTTGKITGNFGLVGSSFTRQQVNPVTNPIPASTRPLVSMPNVEKLLINGQSIQGKACLQTLTINFSNNLEAIRCIGSGKYTPEFYLEKMMDIGVNANFMFSATSASWIDAIKTRDVFTLTFDITDTKGSKYSFNFPQLEVKEANHPDGGGDDIITIDINFAQVRTSPTIVRALV*
phageDNA_00012	6292	6807	+	hypothetical protein	MALKVGIIKSSDVSKWCEYKGADGDVQAEFKVRGIAYKPFQVAIERAGNQISSKGYDVMVKDEDAKLYHELLMDACAAHLIEDWKGVVFAEIVDGKTVESEKPYTPENASKLLNLGDIGISIWLFIKEQAQKIQEDADKDKALILGKSWSSTNTKKRMRRKRRTKSSKSSS*
phageDNA_00013	7310	7609	+	DUF4258 domain-containing protein	MCLTLTEAKQKLRAFARDTSKIKLTAHAKERMKERCISMKQIICCFEHGDITEGPYPNTRGDCQLNVSVRTAGEYITTAVAIKQSENGEFSVVVTTFRE*
phageDNA_00014	7618	8076	+	helix-turn-helix domain-containing protein	MYHYEECGLSNIWLRNGFTIENDEDYGELVSIESVHELHNAIGLFLITQKPDLNGEEIRFLRKELNLSQKNLAGLLGVSETSIRHWEADRGLIGKPTELLLRALYKEHVQGDGKLRSMIESLNHQERTLVPSEISFSYGNNHSWHQTNCEIA*
phageDNA_00015	8181	8861	+	hypothetical protein	MKKILAAGLIGLGLVGCATPAYNYQAIPKNISKPPIGSVNKAFVGDQMLEQGMVVDREVLNVPENIKISFAYSLTSGIYLKTGKNEKGQYFQPFNTVSGGGMVQKNPLADPFKVVMLDTEGKLCVVTVFNAKNCTDKHQATMKTVAIASDNSFQQTLIYSGKFGNKINVGYREFSSNQARPAFNNDVEYDLSQSKQIGYKGALLEVIDATNQDITYKVLKNFNKVD*
phageDNA_00016	8863	9126	+	tape measure domain protein	MSAPQYKPMRESEVCNAIGWVLIALGFIAGFLFILAFGRIEVASYYGKETVWSGVMIATGIGIIFNGFLAGYLFQKVASILRYHENK*
phageDNA_00017	9254	13564	+	tail tape measure protein	MAQESRLVIVIDSQNAERNARNLGNELVSIERKGEFASKSMDSLSVATRALAGHMAGLLTVGSAISKMDTYTGLQNRLKLVTNNQVELNKATEDTFRIAQKTYSAWDSVLQVYQRFSDNAKTLNLTMDDTARLTETVSKAVAISGASAEAADAALVQFGQALASGTLRGEELNSVMEQTPALAKAIAKGMGITVGELRSVAAEGKITSQEIVKALRNVESDVDALFAKTDITIGQSLTLLNNEITKFVGEAGKGSGAAQVLAGSVQTLASNLDLIADGALVVGIGYITRAILMKSAAIKEGMASTLASRQASVLNAQAEYAEATAALNAAKAHLANVRATNAETQAKFGATAATRYAQAQAAVTAATNAQTAAQIKLNTATSIAGRLAKGAFGLIGGWAGVATLGVMGLAAAYSYFNNKAEEAKQKLAEQAKVAEKADEELKKLTGNDKAKAVNDLTTAFNAQNKALEKSSRAVGSALIDIENYARGNREVEKISQEARTGTISYTEAIERLNKIKLPTDLYENLKKQAAQYDDNASKASLSAEKLKLLRVEVKLGGNEAQNAAIQHQKQADALGNTATEAEKATKALQDYQAKQKDSVIDSIYKSGWLDKGYTVAQANAILELQKAKGMSAILSKDEIDSALRNLKIIEEQQEREDKLTEAKRKQTQEIEKQAKLTKRLVGISGQSGIGTGPHLDVRYGGSLSGQKVSNEHLARLQAGGKPLTSYKISSNYGPRKAPTKGASSFHKGIDFSMPEGTPITTNVAVKDIKTWYDSKGGGYVSEVIFEDGVSLKLLHQSPKMQSKVKGGASKGSDKAAGDIQSQLERQQDLQRSLENEVASEVGRINNNRKARLEDVDKANFSPERTAEIKAEINRRADNDIAIAKQALRTKLEDYKEFQKTEEQLLEESFNRKKFNAAHDLELSKFEQKQAVELLEQQKQQELGLLKLAQEQRLFQARLSLLSETQAMQERYRLEREEILKNTKLSIEERQKLIALSKANQDKETRDKVNNAVQNWGGIQADMNGTGEFFRQDQERFSRLNAANDLADSQFAATDLDEKNGLDVLNAHMEAGLIKQQDFENRKTAIIQAAQDQRNQIAAEYAQNAQDIEDKYHQDRLNAQIALGGQMMGSLTSMFGSMFGEQSKAYKIMFAADKAYAIAAAGISIQQSIAKAASVGFPANIPLIASAIAQGASIIANIRAIKDQGFADGGYTGSGGKYQPAGIVHKGEVVWSQEDIKRWGGVGLVEKMRKSANPEAFLNNNALADSVMRRAMMSSSAFIESQKQADIFNQPVQDTQIIYKGNRDTPKLASSGNLDLFHDGKVYFSSNGLVQDRSNLDDVQDFTLGSTSRPQAEMMPSIEPASPTINFKIEVINQVSGATVEAEQLDEQTVRIIVKDELDKQLPRTVPKLVSDQIANPNSTISRSLTENTTARRNRT*
phageDNA_00018	13655	14242	+	Prokaryotic membrane lipoprotein lipid attachment	MKKIIVISTTLLGLTGCAIPAVNNLVRSTNMYQDDVSGNTANLRVYRSNIPMVQFYITYQNNEGEKISKNLITKQISNNLTKYGSMHEPKKLNMPKPTISLNNGEEFFEFKVPANKKLTFRLTSVIGSTTMYSCDVKMDYQLERNGNYELIRLKQIKDFVNPALLTEPSQDEAYCKFVVKEIFEDGKETIIKSIS*
phageDNA_00019	14335	14733	+	hypothetical protein	MYKLKLNPQTSGYGVTPGDDVKRQQMDGGRGRYYIDVKRNSHIVDVNWNLSKTDFNKMMAFWRVYQNKPASFYADLVIDQGTRQQYLCNFIPNSFKTNEVNGNLYRVNAQLEVVQNQPNLNADIALIKDWEV*
phageDNA_00020	14733	15239	+	DUF1833 family protein	MDNEYAKFFFNRKVDVYQLECIELSHPSFMNTYRVVRNDDRGVYVQHKEGSGQVYYEFLPVSIQRSGMLGDLDQTLTVSISGLGDVMPDEFERVIEGQYPDVKPTVNYRIYSSDNLNSPMFYLLGLQLSSVAMNHKAVTFKAESPRLNTTKTGDIFALDRFSGLKGAI*
phageDNA_00021	15236	15598	+	hypothetical protein	MKSHDHLLDRQYDEDHYNCVHFVHEAAMDLYGIDRAEALELFMQPKGKITFLSSRLKLLNPLPMPKEGCIVAFHPRQRNKPPHVGLFRGQKILHLMESGVTYLPEEVVMEMGFNRVSYYD*
phageDNA_00022	15591	19016	+	Phosphatidylinositol-specific phospholipase C	MIKVIYKKDALSEEKTIEQAQTIGQWLTSKYEHMPEHVRIFHTTSNMDHAEISFANEVTPKNAYDLKQLDFLPGTFIVVENPKWVAAIVSIVISIAIAFLMPTPSIAQTTQNTQNQSSSANNELSNRENKIRVNGRIADNYGAGWNTPDLIAVPYKVYENNVEVEHVVGCIGRGHYKINGAYDGETNIVDIAGASVEVFRPGVDIVSGEPYFSLGTEITTPPLTVQHQTSVNGQVLRPADTQSLEGTNYLHFAYPNEILRATANNTDLTTKFVSNDRVEITNASFTFNGQTFDLNGTYSVLSVADDRMTLSNPAAVNANWLKLKELNNQQTAALSPKISSIGEKWIGPFILDNVERSRVLCNFVATNGLYTVSSGGYQAAVNVTIEVEVTPVNESGAAIGNPMLKQIILKGSAKSRQTVGATLDMVTFQGRCSVRARRLTPTPTVTTVVDEVKWQALYGAYPLQSTVYEHETVFRARTYATTGALSVKSRKINFDLQRMLPTYKNGAMTTELYPTSSFADALVSMALDDKIGRRSIDEIDLENIYRTYNDVVDYFGTPLAAEFCTTIDDTNLSFEELVTNLCDAVFCTAYRQNNKLKLYFERPTDNSVMLFNFRNIIPDSYKHDLTFGVMDDYDGLIYEYTDPTDDSRINIYLPDKGAKNPKEVKSVGVRNKWQAHFNAYRIWNKMRFQRKSITFDAAPESELLVLRDRIAVADYRNGIHQSGEVVQQEGLVLTLSHDVDFIAGKSYVIYLQMADGTVDLIPVTPGSAKNKVVLGRLPNGALKLSPDDFVNTIYTVVNDDTKGSLPYLVAKREPVDQFSNTITAINYDERYYLNDKDFIDVPVDDSPIYIRYDQLDINLARLYQMQRGDLPTTGEISFVVESGALVSSSSSYRPETRFVYKFDYNSSPPKQEFIAPAATELPAIDTGEFPPDLVVNLTIKGAVVGRGGDGGLPHLAFGAWESDPDYNFTKTRRDGFQGAPGLLNRHSKLNLIIDGGTLARGGSGGGATPSGIYTGLSYGVQGIPGGAGAPFGRVMTGQPISSDSQDWRWYFGSYFNVLKITDAEASVPGKGYRTQNDRYGSPLSGDGGNWGERGTKSTNDGTWNWKYHGTTEGQPGPGGPAIVGVAPLTTQLINGGKILQTL*
phageDNA_00023	19084	19473	+	Ammonia monooxygenase/methane monooxygenase	MEPVSTSGFTALLKLYGIAIMVTLAVGLVAAVVLMTRMPRSPQEWAVGLICTVVSSLAGGSFIIVKWGLHEWVTDVWGMIALGGFFFVCGLPGWALVRWIFNFIDKQEGKTIVEVIKEFKKARKDIENS*
phageDNA_00024	19473	20060	+	Endolysin	MPPSGGFLHLKETEMNIEQYLDELIKREGGYVNNPADRGGETKYGITEAVARTNGFKGNMKDLPLDVAKAIYKKQYWTDPRFDQVNVISSLVAEELLDTGVNCGTGFAKPLLQRALNLLNNQGKAGWPDLTVDGIYGPATLNALKTYLAKRGKDGEKVLVRVLNIMQGQRYIEICERNPSQEQFFYGWIANRVVI*
phageDNA_00025	20542	22860	+	TonB-dependent receptor	MKPLAALPLIFIAIQQVQAEERIAILKPLVFKANALKEKNYVVDKEKVESSNTIGDALKNITGIQSTSFGPNAGAPVIRSLSGNRVGVIENGEFINGMNAFSGNINIPFDPIFIEKVIVNKNTDNIRYGGNAIGGSVQIESGLIPKKIEEKPNKLDIVFRKGFNDFDVKGFNFNINDQKNWSTNIRYSEYEISSYKIPGNSKAKLCEDQIFSNSGGINSALAASCQKDSRVQHIYNKSSQPYIDKFMTENPDWADGDFSFYTDKPTSIWGGKTYINPKNPEYIPNTPQNTIKKINTDVTPNYFKKLGNSYAQNENIGFGTTYFFDKGFIGLSADKKKSEYGVPGFSLQNQSFADSYETLPVGVKIDQNRFVLNSNFIQPISSAEEISLNFQQLSNKSGEYVGTAKANEYKIDNQLIELIMKQSSFKGLDGILGFSLKNRNIEGSGTQRYLPNVSTISKAIFLQEELNIKQFTLNTGYRFERIEHELQDNDFKLARNASNSRIENRKYNLNQYFIGGEYKVNNFINLKVDYGISERAPEVNELYSSNVHYSVMAQEEGDQNLKPEKSKSLELGMFLNWNNWVMQLVGYQMDFENYMYLSHSGVAVQNRLPLKYWKQTDTDVKGFEIDLIYDFNLAHIGNIKLGGLADFVKNKAVNPTDIRLANDGVYLPNMPTNRYGMFLEWRNDSWKGKISSIYYDEPRYLGKNVIQEVPLSGYNLLEFKIDKKLKIKNASFDIFLNGTNLLNEEARPQNSPLKYIAPLPGRAFQLGITMHI*
phageDNA_00026	23142	24527	+	Phosphoethanolamine transferase EptA	MALNVFKFKKICKDVTLLNFNLLLSIWLGLFLNIGFFKKIHQLTPYNGIKSVLFLGATLVILIAAYNLIFQLINWKWTAKIFAILLIFIGGFSSYFVNTLGVIISPDQIQNMVQTDVSEFTDLISLRFVLWTVFFVILPIFLITQVKFKQEKASRLLLKKVFSLVASFAVVGVLLFTYYVDFAAIFREHRDLKGMISPQNSISSLMSYYHKKAPKKNLPLVIYGQDAHQVQQVQKNLPKLMILVVGETARAESFSLNGYAKNTNPELSKQDIFNFSQVSSCGTATAVSVPCMFSGMPRVDYDEQLASHREGLLDIAKRAGYQVTWIDNNSGCKGACDRVEQYQIPENLKKKWCKDGECYDDILIDSLKQYLATIAKDDDRPRLIVLHQVGSHGPAYYKRAPEAYQPFKPTCDTNAIQGCSQTELLNSYDNTIVYTDHVLSQMILCTRQISQNPYPIRVLPS*
phageDNA_00027	24489	24935	-	Transposase DDE	MVGRVKLYISALQLENGELLLVVSPQFNANAIQDYALRWEIETLFSCLKGRGFNLENTRLTDPRRVKKLIAVLAISFCWCYLTGEWQHDQKKAIKIKKHGRLSMSLFRYGLDYVQMAIQRLIGFGKKEEFKEILAILRRQNPDRIRVL*
phageDNA_00028	25010	25579	-	TnpA (transposase)	MTHLNELYLILNKYLKWNKSHLKCFALIMLVIILKQTCNLSSASKALPIKCLPQSFYRRMQRFFAGQYFDYRQISQLIFNMFSFDQVQLTLDRTNWKWGKRNINILMLAIVYRGIAIPILWTLLNKRGNSDTKERIALIQRFIAIFGKDRIVNVFADREFIGEQWFTWLIEQDINFCIRVKKTSLSPII*
phageDNA_00029	25654	25977	+	Phosphoethanolamine transferase EptA	MINTLKEISKYQTGLWYLSDHGESTGEHGLYLHGSPYAIAPSQQTHVPMIMWFSESWKQHNLAQVNCLSQQTKQKLSQDNLFPSLLSLLDVKTQVVNNKLDMLSQCK*
phageDNA_00030	26288	26776	-	SEL1-like repeat protein	MKKIFLAALATIGLQACTTPYQEMGASGGVEATIIDDNVFQVRASVNGYTHKSIANQYALRKAAEVSKSLGCSYFSAINNTSQSYNQNISKVDAGLKTTPNGVYYVSSAGTQYKLIKPSRNNTYVCFNEKPNTVLPGLIYNVKYVLGSPMPTGKFKVPNSWR*
phageDNA_00031	26866	28239	-	Recombinase	MAQHIKFTKSVIDSIPLSEEKQIFYRDTVTIGFGLCVGKTKSYFAEKKMPNGKSKRKVIGKHGVYTLEQARTEAKRLLILMDEGVDPVKQKRDLRASAIQNDALQKLVPTLSEAYQYYKLRKKLAETSLIAYDGCIENYFSDWKDLKLDQITSAMIIDRHLKLSKASPSRANLASKFLHALFNHTISRYKDESGNKILNIKNPVVIVKEEKAFNKIKRRKGHVRADQREAWALAVATTYWMGEQNNDFRAYTNQDFLFLLALTGFRRSEAESVEWKNVDLQFGTIKIINTKNHEDLLLPMGDTLWHIMRERKKRAGDNKYVFTDRNGVSHISDRRAARDKVTENSGIEFTFHDLRRTFGTIANSLAIGSYTIKRLINHTTDDDDNDVTDGYIQVSFDDLKKAMNMIEDVIISEPVKALIKSRLYFEKNESRNQAQELINHHIQVLDNFKMAGQILDF*
phageDNA_00032	28674	28937	-	DNA-binding protein	MSRLTKLDRMTHAEKEAAKKEFWEAADNQTFPPETVAIVMHVSLPWLQKKRCEGGGIPFSKPHKRQVNYMKSDVLAYIEQNKMAHTA*
phageDNA_00033	28938	29660	-	CdiI immunity protein	MSESTLWAVAMRPEGYSPFKQTPAASKEIAERAVERYRKMHEKEGNNFFLEIFDDVIKVQKWHGSRKDHIKNLFYVESWFSEPMYQCFDLKTAERVFKFDEIVICYKKGSAPLVTKSFDEAKLFYGSSETGFKYQIQPIEPPENLFNWFHPDIELFDTIEEGAEAYTREQWAQLQMNLRVEIETQLLDYDEIPNIPEDAVVWPNWKPEPPEQGLFLIAAFDSEDGPVLWWANPKAESKEK*
phageDNA_00034	29657	30064	-	hypothetical protein	MTGNERIPFESQFKTTEIFKRESAIRKNDILAFSETMNGYFNIVTNDAWQLWNKAKAETVPDTPTPSVTLTCAELKEAFDFGAPDGEKDQFQMETEMTIKWLQDGYDGEGYYCWYADLPEEGCIKLGVSESGAEG*
phageDNA_00035	30065	30280	-	hypothetical protein	MLGFMYYLQLLGYVIVRQGMDQAMFLTKHYAVPVAWRRITIDYHNRLNKPAQQLYKEFVEWTKEEYAEMVA*

phageDNA_ 00036	30318	31250	-	hypothetical protein	MKILNKVEAKLAWANGELLLVNNTERNGWEPFNPYDFGFDVFDKFEFQLKPRTIFIGEFEVPEPLRVAPEKGSTCSYPSPTVELGVQQFKWNGSKGQLRMLQHGQVHSSFDNAFAHCCAIIKISGGEFAEDILKLLNKPTEEVEEEKPSESHAESLKVKNSKPEVVEKAQTAE PAIESETADSEYQKKLDTLLQRVKDSKTPDEVNAVYRYTRTWSDKQMEPLLLATHKRLEELEKSKAQATEPPSLMVQIQNAPDLTTLDALEIDVAARDPQIQSRLMDFVKKRRFELENPTSTPLQEAEPDYLLGDGF*
phageDNA_ 00037	31247	32383	-	ATP-binding protein	MRIRKMALPIITADQTLLVQAIIVYLYADPGLGKSSMGFTAEKAISFDFDRGAHRTGELRRGAVVQVQQWSDVANLTPQDLAPYKTVVIDTVGAMLECIKTHLLLTANNRQKDGSLKLKAQGLANQTFKQYINTLISLGKDVVFIAHASEDQNGDQIIYRPDLGGKNRNELYRIAD LMGYLTTVTTGEGKNARVINFKPSPTHHAKNSGALGGETGEVWVPDLKAHPTFLADLITQAKDHINTLTPAQLAAAKAQEELENWKQSCEEAEHAGDLNQLTESLDKEHMYYQNMRQAMLMRAKALNCTFDKQRGTWISPPEFNGISDQQRDELQNFIAERGLDVKTVCE HFGIDALIQIEAAKLTAVKQEIETLAKTGMTA*
phageDNA_ 00038	32380	32703	-	hypothetical protein	MSNFKKHPDGYMSFLGRDDKGLYSVRIGWQVYASNANGSVLYKVKDGVKTPLNVFRFQTSYPKVWNELTQEIDFQRRKQLAIKLRETNIPTYDRKAYKQKRGFTGSR*
phageDNA_ 00039	32696	32986	-	Ubiquitin-Binding Zinc Finger	MKDYNCPTCKKMIPVDRSKIKAGDEVSFCRVTQSSKSARFSSREGIVNCREGDVVLVKYRKEIIPLNIRDVSPVDAPSPLTYAFVGTCECKEAEHV*
phageDNA_ 00040	32986	33426	-	hypothetical protein	MNVKTFSNKHKVTGVTAIAVLVALSSCEYRTANSSVPSNYSYESEQVVASEYELLAVKKTGEKSGEAVIRIDGFKLNVSFDFDGVADSYGVAGSDFTTAEITNLAIDSVTDLSGKSFNDFTNHDDHKNINILLAGYIDRNNWLEAA*
phageDNA_ 00041	33635	34138	-	Actinobacterial Holin-X	MAKRYLPFYNNARFIALVLVGLFAIFSIIFKYLELNITINLVQFSFVLLLPLSQIYLAYKGMLDALKLDGLNQSERDRLTSTVDIRSKSSLYVAMLFIILVFSMYILNLLGLLSAKHLLALILSVGLTSIFSFFLAWSDLREISLLEKTLKDRKESREAKAKVLSNK*
phageDNA_ 00042	34140	35192	-	hypothetical protein	MNPTQYFRAIREEEIMSKTVVKDKTVHYKKVDFLKGANLGQLLKAQLLDKDSFYYKAINRQQFVSATKDDFILINHASSHQSMFFGELIIVESGKAQAVLKIDSDDATEFPIKTYLTDDLPDDEDGVDATEVVRKEFIDSVLYFGVIDNHVAIIQSRSLTARTLESYLGWLLGEAAK ALPENSALILKDAPNPTVKQKLESTPAKTISISSGIGSTELQPVHTVESSIPAKIDYKIEDNVVDVLKSAFGVDLENLKLEDGLDDANLKLKLTLTYNRKTSKSGQKVIDTVASSMRHNDDYVITLEDGTKVTADNLKMSGKISVETINNKVYNDGLKVQLYNWMTTNINFGD*
phageDNA_ 00043	35199	35414	-	hypothetical protein	MDNSKRPINQIIARINDAAKHGEALVLTAEEVKILSKDIGDKVFIPVLTNEQVVQLVKEGKLGHKINNTKD*
phageDNA_ 00044	35429	36193	-	LexA (CI repressor)	MNTLAERLRYAMEVLPPKKIKGVELARAVGVKPPSVSDWLSGKSKTMEGENLLRASKFLNVNPSWLASGTGEIQSSTRDKFKQLDIEAFKKKYNISDSDEALLFSTIIEKPFTPSSKRWVPVKAYSKMGMDGYFTDMGYEGNAGDGYVPTHSAGPRAYGIKGTGDSMFPAIR NGWYVVCDPDADLVPNEFVQVCLKDGRCTIKEFVGINGGVLSLLSVNGGERFFFEMDEVESITAITDIVPPSQHRQEHPYSH*
phageDNA_ 00045	36302	36553	+	Cro	MNDAQLIDKLGGVTAVARLLGITPPSVSGWKAIPLDKKIRLAVIAEDLGLTTRKELFPDNYQDIWIELRPQTTKSRNLGSLTA*
phageDNA_ 00046	36564	36884	+	Bacteriophage CII protein	MSKVSNELPASASNNESLILQALNTSNQRQVAEKVGIDASTLSRMKNDKKNNGLTEIEFISSLLTAIGLKVVPESDVYCSPEIAEATRVYLAHAFTSPEYMRILFK*
phageDNA_ 00047	36841	37215	+	hypothetical protein	MHSLHLNTCGFYSNKKPLPAGTGVVRHSNEVDQMNTNNLSEQPIELNSPDFLIGDVVVLTKECRSFKSNDLFEVKNKTLTRLWTIKSENHLILVSSKEIRTATVAELNAKRRLTKAEQALAEVS*
phageDNA_ 00048	37212	37511	+	Iron-dependent Transcriptional regulator	MNSQFKYKPEYKQTQEIQSFFDPALVILNELHDRNRKNLRAKGYDENNAAITREEFSQTMAQRFRINQWLAGQIVNSLANADLVQKFGGYVKPKVGVHE*
phageDNA_ 00049	37504	38385	+	Bacteriophage replication protein O	MSNFVPNSFQVPNAFVDEVLNKISDAACKIYLVICRKTRGWNKEMDSISLTQFEEITGKSRPTVVKCLNELIKVGLVVEQPSTIHGNTFKLGNDTSVGLVLKFPSKNFLLPEIYGQTSKNSLPLLVKNFNYTSKNFLPLLVKIFNTQSITIKNNSQSNKKINKKREPVSEKPKSEKPT EFNPRLVELPACVDPELWNNFVDMRISIKKPLSENAVKLILKKLISFGPLANQSLENSIIGNYQGVFEPRQNQIQENPQSHNVPEEPGYFTQMYAESNRSNVIDVTPVSQDFGGY*
phageDNA_ 00050	38388	39188	+	Replication protein P	MNELAPFESFLKELIAAYRTKYAVQFNKNFPVEGKNAVPMQIVEQQLAKALVGVTPNQLQRGLALFYASTNTYMPNFAEFRAMCMGDDWWSAEKAWVKACEYTQISQHKKVTLPDGREQNQEITTLTKFVLDQVYSLIQDGEMYKAKMEFIKIYDEYKAEAQLKGKTQAWY QEPILLAQKNEQKVHKPVSNDEAQKHLQSLMERLKINGRKPVPVQKLKAKEKEPELKQELGPDPFDNPHEYAEMCRREGMPIPRNILKLIDGANI*
phageDNA_ 00051	39185	39319	+	putative phage replication protein	MNKFEILAWGLLISFFTAAISGAVVWWWLARKELDEKGASHESN*
phageDNA_ 00052	39288	39728	+	Ribosomal protein L7/L12 C-terminal domain	MRKEPAMKATKLIRDKGLQYAKEIVDSAPDNATEWNEGYEFQCGQSVEISPADREKYFVDLVELKRLVESLKIINDLGGVEKLTPAFITTDKHVGYTHVRMVGNGRLSFLDDFCDFIPDGSISIKRVMTAIRDHESIYGGGESHAN*
phageDNA_ 00053	39718	40140	+	hypothetical protein	MPTRYNTGEYSYDLEYHYGDMSASMEMLRARLIELLTPHLSGRYVKWREAYFTWFTKCGGDSGWMFCVGPHEFHIDGALRRYYSGSIDITYNQKDRYFLVGEKKKVKCKACKGFGFIRDDGWGHIDKCEMCDAEKGASHE*
phageDNA_ 00054	40133	40357	+	carbohydrate binding domain-containing protein	MSEFEGKSGKWAWEIQKEQQAKVEELQKRLDGALKETQYALQYVEEDMRGNHEFLQMAMIRTLKAIEQVLKGGA*
phageDNA_ 00055	40357	40758	+	DUF559 domain-containing protein	MSSVSIAEYRKLFPIKKNKKRRSAKQVARQPSVGEVVLATHLRACKIGFEQEYKFHPERKWRADFLIKGSKILIEVEGGIWSGGRHTRGKGYLGDMEKYNSAAMMGFTVLRFSTEQVKSGMALKQIELLIKGK*
phageDNA_ 00056	40769	41521	+	hypothetical protein	MLVEKFDFIELLRLAIAQGKAEGKKISKDVVLGELALLSPAAKLWATVLIEKVDFERIAIITPAQKQTETFYSKYDFNFQTERRIEDIPGKVEFVRGEIKSGNFFRARNKLAVEIHKEMVKKKFTPTNAQGDLTNLAKGMAEIILRGHVFVKAMCGVCQGLGKIETFGLNGFPNGAR FCEKCNGTGKRPYTLKEKMNIAGIDATKTAYIKSYQKFELFGESIVAEWENEIRTRISRSFRFELPDSQETYA*
phageDNA_ 00057	41943	42398	+	hypothetical protein	MTQYLMFAENIYNKIKDEELFSHDCIENMNLLMTCIRREIKGTEFKLKYNFIDFVELFSRPLDECKVKIDVSLIPPHNSEGEYILWLAGLIEKITEGGPKPPPPIKKFIPEYMSLKFELDFLPLNEEKIQNEGKEITDYFNSKLYKATFKK*
phageDNA_ 00058	42460	42894	+	hypothetical protein	MDSTEYFWLTRKKEPKTKPKSRPLPKAKQKYLEAEATLKEELEDLAIGFESKFQPIHTKHWRFDFHIVKLRLLIEIEGGPWSGGRGGKLSNKAWSLNRYDHAEEMGYKIERFHPDSILSGYVINWIKSELARIEDGADQTISTD*
phageDNA_ 00059	42863	43504	+	hypothetical protein	MEQIRPFPPTDFIDQAEEEEAIRLTPAPDLKKWVVANYLTIGGPIYNPDHDHIAELLHDNDEFLAFAWASSAYKSKQAMVLGQCEKVMFNVGGWRKARQEQQMRDWFGFVPTYLITVDASFCERANDTEFCYLLEHELYHIGVMRDEDGEIVYSDSSGLPKHYLAGHDVEEF IGVVKRYGPSKNVKRLIEVAKNPPFVSNLDISKCCGNCVIN*
phageDNA_ 00060	43563	44033	+	DUF2280 domain-containing protein	MATLKEPVKIFIVQSLACRDTPQEVAELVKQEFGVDIDRVQVATYDPTKVAGKNLSKKYVELFEKTRDEFDKGLIDIPIANKYYRLKQYQRQLEKTRNVKTALKILEQAAKDIGGQFTNRQEITGKDGGPVQTVNSEIPVPMEDYLKARREVLDEY*
phageDNA_ 00061	44023	45450	+	Terminase large subunit	MSTDAARDKAIRIEAQEDLYFFTRYMFKERRGYKWMQNWHHLEICEALMKVYRGEIKRLIINVPPRYSKTEIAVINFMAWCFGKNPDCEFIHISYSAMLAANNAFQIRTLVQEEAYRKVFPELTLRDDSKAKDFWRTSQGGVCYATGTGGTITGFGAGKLRKGFGGCIIIDDPH KAHEASSKTIREGVIDWFQNTLESRTNSPDTPIIVIMQRLHEDDLAGWLLGDRKDGVPVAGGNGEVWEHLCLSAIQEDGSALWPAKHNIQKLRLMEQAAPYVFAGQYRQMPSPPAGGFFKPDNIQIVDALPADVVKQVRAWDFGATENEGDFTVGVREALGADGFTYIVDV TRGQLGPDNVNKRLEQTAKIDGKKVSVRLPQDPGQAGKSQASSFVKLLAGYSVIAKPISGDKLTRAQPFAAQVNVGNVRMLKGEWNKDFIDELRHFPNGTHDDQVDAASDAFNELHEGFEAFFADMGFAR*
phageDNA_ 00062	45447	46898	+	DUF4055 domain-containing protein	MSDVTFQHAEYVKNLPYWQKLDDVCEGEDAVKAKGEKYLPMPNAHDKSPANKSAYEAYLTRAVFYEVTGTTLNSLVGAAFATDPSFKFPPELAHLERNANGAGLSTYQLAQNGIRHLLKHYRCALYVDYPDVPPARNLAEFKAQKAYPMIHLLNALDVVNWDSVMIDNQK KLCLVVIREFKSERGADGFSKTEQEQYRVLRLEQEGNGEYIYSVQVYTKGEKGNWVGGEKKFPTDYNGNFWTYIPFTFVGAIDNSEEIKKPPLLPLANLNLAHYRDSADFQESVFYMGQPQYYAKGVNWEWYDQAKKRGIYIGAKVLLPLPENGGLGIVQADPNTLAREAM KDKWEKMKEMGARLIEKGSGSKKTATEANSDDAVQHSVLSLCVVNMNEALSAALRWAAKFVTPNVDVLTKDDLMFEISQEFNKQGYLAELARQLFEAALQGRSSFKSWWEYNQTGMFPKQKYEEELQNVEAEQDGTLNQKVE*
phageDNA_ 00063	46900	48003	+	Minor capsid protein	MATDIKKLFEVLTQHQAYLYRASSKTVNELLALFNDDTSKMLSKLRDLLDELNESEKVALAGGKYTTSNLREIRDLIAQWFASVNLALPEAFAVSATALAVYEANYVAKLYGAKINKPDGEKLFLSAKKVPLAGGALVDDLLSRIAESARQKVEYAIRDGINSGKTNQEIVQRIRG TKRLNYEDGILNGTKTDIERTVRTVRSHVANQAYLNSFNQIGFEYVRFVSVLDGRTSKLCASLDGSVWEINDPAKRVPPLHPNCRSILVPVEKDGQLVGERPFVMDERRVKDIPKEERSQLIGQLDANTTFKEFFKKTDDFFQREWLGPKRFKLYKDGKFDFDKFFDPEGRFY SLDDLRKLDEKAFKKLGL*
phageDNA_ 00064	48012	48440	+	hypothetical protein	MLYFLKHQNLYNMKTIAFVCLTLISITCLAEPSQKYLKEYDRLSEALESAMANAYSFDPATGQVKQATQGLEAKNNLCRAAQAKLNLTTFLKDNLEESKELYKSIDGAETLDKNYLSGQQQEQQNLVSNLKKDLVGTGFNCE*
phageDNA_ 00065	48539	48781	+	hypothetical protein	MSESKVRHLVLKRVSDKSSHLALCDEETGIPLAGLTAVKMNCSVFEGPATITATFDVGGPQGIRLVGDEPRQKVWSAKET*
phageDNA_ 00066	48999	49190	+	Occludin homology domain	MPSPIIQYFQYEHLPEHLQQVSKPIGDLARQMDEQLPDGPEKSTGLRKLLEAKDAFVRQALSK*
phageDNA_ 00067	49304	50071	+	Telomere regulation protein Stn1	MKLKTVTIDGKVYAEVDGDKPIYIHDDGKEMPHDAPHSVATIARLNNEAKTHREAKEAAEKALKAFEGIEDPAAAKKALQTIQNLDDKKLVDAGEVEKVKAEAIKAVEEKYAPIVAQRDALEASLHKELIGGGFARSKYIQDNIAVPVDMVQATFGHHFKIEEGKVVAYDPNGEKI YSRVRPGELANVDEALESLVGGYQHKDLILKGGKGTGGGFQGGGKGGAPTGMKRSEMSVSQKADYIKEHGNDAFLKLPN*
phageDNA_ 00068	50449	50619	-	hypothetical protein	MRPNLSPNFRIPRVNALRPSTTILPFATMSGLLEIAAFKEAKLYSNRPATNASARS*

```
LOCUS       phageDNA_Annotation    50929 bp    DNA     linear      23-JUL-2021
DEFINITION  final.dna (50,929 bp).
ACCESSION   phageDNA_Annotation
VERSION
KEYWORDS    .
SOURCE      Acinetobacter baumannii bacteriophage
  ORGANISM  Acinetobacter baumannii bacteriophage
            Unclassified.
REFERENCE   1  (bases 1 to 50929)
  AUTHORS   Kim,K., Islam,M.M. and Shin,M.
  TITLE     Direct Submission
  JOURNAL   Submitted (23-JUL-2021) Dept. of Microbiology, Kyungpook National
            University, 680 Gussaboseong ro, Daegu, Daegu 41944, South Korea
COMMENT     Bankit Comment: ALT EMAIL:maidulbau6923@gmail.com
            Bankit Comment: TOTAL # OF SEQS:1.
FEATURES             Location/Qualifiers
     source          1..50929
                     /organism="Acinetobacter baumannii bacteriophage"
                     /mol_type="genomic DNA"
                     /strain="Acinetobacter baumannii bacteriophage 1656-2"
                     /isolation_source="Acinetobacter baumannii 1656-2"
                     /host="Acinetobacter baumannii"
                     /note="[cultured bacterial source]"
     CDS             192..857
                     /codon_start=1
                     /transl_table=11
                     /product="stress-responsive nuclear envelope protein"
                     /translation="MKIIYTRIAAAAALETGIIANPDYYENPNLKAKEVIIYGNYPKI
                     QKDYESLEVPVEVRKLEVPQKTTLATVNVAVGITPELQAVMDDAKAECEKVVEENTQL
                     KQKIAILEQAGGNQSELLSENSRLKDAAVLADKALKDAEAQVVGIKTEFEAFKNDIPA
                     MQARIAELEAGKAAENPATETAANDFENWSNDQLKEYLASKNIGYKPSATKAELLKLI
                     PKE"
     CDS             862..1251
                     /codon_start=1
                     /transl_table=11
                     /product="T4_neck-protein"
                     /translation="MSFITVDDANSILGSDFAPDSDKARLVKLANVWMKNRIGFVPDP
                     IDPLLKDAACEIIKGILAKVIYNGKEQQLKRKKVKADSVESEKEFQDGSEAISSFEQI
                     AIDFIDSLDLKDPNASFNGFGIPLYRA"
```

CDS 1208..1621
/codon_start=1
/transl_table=11
/product="hypothetical protein"
/translation="MQVLMALAYHFTGHDMGLRDEIQADIAEAFNDDLADAIHTFTCE
RISKTNWDPKTETYVEVKENYSGRGVLFGSYSQYEIQTLGVLATDKKATVLQNEVTMT
PKIEDEWLTALGSFRVINIQQDPANTIWKCQLRKV"
CDS 1600..2034
/codon_start=1
/transl_table=11
/product="Peptidase"
/translation="MSASKSVGAKMVNPDYVPEWYISPFQHVQYTLARNQLHMDLLFE
DMDKADQFLDMGADAQVSTFSDGAYAIVQIGDTADKDRIQVYGLLLHEAVHVWQKIKK
LMGEREPSSEFEAYSIQAIAQDLFKMYEESEVNDGMEGEKAN"
CDS 2006..2374
/codon_start=1
/transl_table=11
/product="hypothetical protein"
/translation="MGWKGKKPTEFSFDVSKAAEDQVKHIVMDTVQSLVNLSPVDTGA
YRASHIVSVGSADFGVREPETNPINDAAIQAMKIKLGNLVYIQNNKAYGPRLENGWSD
QAPQGIYGLTFNFISQKYGG"
CDS 2382..2774
/codon_start=1
/transl_table=11
/product="putative Electron transfer flavoprotein subunit
beta (etfB)"
/translation="MTLEQTRQAIIDRMQSFTGIAQERIQYPNAPDFTVPTKGVWCRL
TIAGGPSFTSGIADKPCTRRTGNIMIQCFDRLHTGEKAVTVLSDALLAHFEYFTIEHL
ECLNGQSIYAGKDADFIQYNVSIGYKVN"
CDS 2776..2994
/codon_start=1
/transl_table=11
/product="hypothetical protein"
/translation="MSCMLTLEEIEIKRQELERHLEDVMSVELSKWQSENKLCVSDVN
IRLANVVSLGGPKHNVVTGVSVDLDNEL"
CDS 3103..3624
/codon_start=1
/transl_table=11
/product="bacterial SH3 domain protein"
/translation="MKKSTLGWGAAGLVALGIFGSGNDNSPKQTSDSENAQSAVEEVI

ESKYINTNSLNIRDKPNGQVVGKLGRGEKVDIYEMKGNWARISLNSSSPQWLSTKLLC
ETDGCFKQKSRSTTSNNYQALKSHPHHSERKQKKTYYDSDCSCAVVDYCVGPRGGHYC
ITSGGNKRYKPRY"

CDS 3721..4074
/codon_start=1
/transl_table=11
/product="hypothetical protein"
/translation="MSNFVFKRGDTFNLNLQLVDMDEALQYPPDDVRRAIDLTGYTFT
SQVKALADGATVATLTCTALSQSTQKGWLNIKSSASTATWPLGLCQMDIKAVVSGTTQ
HTETLTFQVIDGVTA"

CDS 4074..5252
/codon_start=1
/transl_table=11
/product="putative tail fiber"
/translation="MANLVFKFSWDHRPFPYNASQGKRQFMLPFASGIPNLAPNFSQV
VGTAAISQGGTGATTAAGARANLGAAASGVNSDISELKGLTTPLSISQGGLGADNAQT
ARMNLGLGTAAVLASTTSQYDPTPGRALRVGDWGIGAEGSRVSDMVAPLNNGFFRTDD
TLTNDTGNSIGPYGFFLHCTRRSMGLYTNGSHSFQLGKAASYSALKYRFNNSGTWSNW
FNLLTAQNTTTDGNGFIKAASPVVKLFQSHIELNNDAAKQPITFDKLGTGDYLIKGSL
GFAQEGWYIEVPKDANGNTVVAVEYSTLENGDLSIKTYKRKFDVEKAAIVADLENPLD
IPEGRWIDIRLHEEPEPEPEEPLSETPVDFQPTNLSQAVAAAMNGVEPPEISETDETL
"

CDS 5305..6222
/codon_start=1
/transl_table=11
/product="hypothetical protein"
/translation="MSSGAKIRLYACEEAVLGTTPANPVWYTVRRVTDSLTENVTTED
SSEVVDSRFRQGAVVTEAEVTGQLEFELSLGTFDLFLNVLAFNNWAANALSFGGGVRK
SLTLVKVFEDIGQVFIYRGIQVNTGEMTIQTTGKITGNFGLVGSSFTRQQVNPVTNPI
PASTRPLVSMPNVEKLLINGQSIQGKACLQTLTINFSNNLEAIRCIGSGKYTPEFYLE
KMMDIGVNANFMFSATSASWIDAIKTRDVFTLTFDITDTKGSKYSFNFPQLEVKEANH
PDGGGDDIITIDINFAQVRTSPTIVRALV"

CDS 6292..6807
/codon_start=1
/transl_table=11
/product="hypothetical protein"
/translation="MALKVGIIKSSDVSKWCEYKGADGDVQAEFKVRGIAYKPFQVAI
ERAGNQISSKGYDVMVKDEDAKLYHELLMDACAAHLIEDWKGVVFAEIVDGKTVESEK
PYTPENASKLLNLGDIGISIWLFIKEQAQKIQEDADKDKALILGKSWSSTNTKKRMRR
KRRTKSSKSSS"

CDS 7310..7609

/codon_start=1

/transl_table=11

/product="DUF4258 domain-containing protein"

/translation="MCLTLTEAKQKLRAFARDTSKIKLTAHAKERMKERCISMKQIIC

CFEHGDITEGPYPNTRGDCQLNVSVRTAGEYITTAVAIKQSENGEFSVVVTTFRE"

CDS 7618..8076

/codon_start=1

/transl_table=11

/product="helix-turn-helix domain-containing protein"

/translation="MYHYEECGLSNIWLRNGFTIENDEDYGELVSIESVHELHNAIGL

FLITQKPDLNGEEIRFLRKELNLSQKNLAGLLGVSETSIRHWEADRGLIGKPTELLLR

ALYKEHVQGDGKLRSMIESLNHQERTLVPSEISFSYGNNHSWHQTNCEIA"

CDS 8181..8861

/codon_start=1

/transl_table=11

/product="hypothetical protein"

/translation="MKKILAAGLIGLGLVGCATPAYNYQAIPKNISKPPIGSVNKAFV

GDQMLEQGMVVDREVLNVPENIKISFAYSLTSGIYLKTGKNEKGQYFQPFNTVSGGGM

VQKNPLADPFKVVMLDTEGKLCVVTVFNAKNCTDKHQATMKTVAIASDNSFQQTLIYS

GKFGNKINVGYREFSSNQARPAFNNDVEYDLSQSKQIGYKGALLEVIDATNQDITYKV

LKNFNKVD"

CDS 8863..9126

/codon_start=1

/transl_table=11

/product="hypothetical protein"

/translation="MSAPQYKPMRESEVCNAIGWVLIALGFIAGFLFILAFGRIEVAS

YYGKETVWSGVMIATGIGIIFNGFLAGYLFQKVASILRYHENK"

CDS 9254..13564

/codon_start=1

/transl_table=11

/product="tail tape measure protein"

/translation="MAQESRLVIVIDSQNAERNARNLGNELVSIERKGEFASKSMDSL

SVATRALAGHMAGLLTVGSAISKMDTYTGLQNRLKLVTNNQVELNKATEDTFRIAQKT

YSAWDSVLQVYQRFSDNAKTLNLTMDDTARLTETVSKAVAISGASAEAADAALVQFGQ

ALASGTLRGEELNSVMEQTPALAKAIAKGMGITVGELRSVAAEGKITSQEIVKALRNV

ESDVDALFAKTDITIGQSLTLLNNEITKFVGEAGKGSGAAQVLAGSVQTLASNLDLIA

DGALVVGIGYITRAILMKSAAIKEGMASTLASRQASVLNAQAEYAEATAALNAAKAHL

ANVRATNAETQAKFGATAAATRYAQAQAAVTAATNAQTAAQIKLNTATSIAGRLAKGA

FGLIGGWAGVATLGVMGLAAAYSYFNNKAEEAKQKLAEQAKVAEKADEELKKLTGNDK

AKAVNDLTTAFNAQNKALEKSSRAVGSALIDIENYARGNREVEKISQEARTGTISYTE
AIERLNKIKLPTDLYENLKKQAAQYDDNASKASLSAEKLKLLRVEVKLGGNEAQNAAI
QHQKQADALGNTATEAEKATKALQDYQAKQKDSVIDSIYKSGWLDKGYTVAQANAILE
LQKAKGMSAILSKDEIDSALRNLKIIEEQQEREDKLTEAKRKQTQEIEKQAKLTKRLV
GISGQSGIGTGPHLDVRYGGSLSGQKVSNEHLARLQAGGKPLTSYKISSNYGPRKAPT
KGASSFHKGIDFSMPEGTPITTNVAVKDIKTWYDSKGGGYVSEVIFEDGVSLKLLHQS
PKMQSKVKGGASKGSDKAAGDIQSQLERQQDLQRSLENEVASEVGRINNNRKARLEDV
DKANFSPERTAEIKAEINRRADNDIAIAKQALRTKLEDYKEFQKTEEQLLEESFNRKK
FNAAHDLELSKFEQKQAVELLEQQKQQELGLLKLAQEQRLFQARLSLLSETQAMQERY
RLEREEILKNTKLSIEERQKLIALSKANQDKETRDKVNNAVQNWGGIQADMNGTGEFF
RQDQERFSRLNAANDLADSQFAATDLDEKNGLDVLNAHMEAGLIKQQDFENRKTAIIQ
AAQDQRNQIAAEYAQNAQDIEDKYHQDRLNAQIALGGQMMGSLTSMFGSMFGEQSKAY
KIMFAADKAYAIAAAGISIQQSIAKAASVGFPANIPLIASAIAQGASIIANIRAIKDQ
GFADGGYTGSGGKYQPAGIVHKGEVVWSQEDIKRWGGVGLVEKMRKSANPEAFLNNNA
LADSVMRRAMMSSSAFIESQKQADIFNQPVQDTQIIYKGNRDTPKLASSGNLDLFHDG
KVYFSSNGLVQDRSNLDDVQDFTLGSTSRPQAEMMPSIEPASPTINFKIEVINQVSGA
TVEAEQLDEQTVRIIVKDELDKQLPRTVPKLVSDQIANPNSTISRSLTENTTARRNRT
"

CDS 13655..14242
/codon_start=1
/transl_table=11
/product="Prokaryotic membrane lipoprotein lipid
attachment site"
/translation="MKKIIVISTTLLGLTGCAIPAVNNLVRSTNMYQDDVSGNTANLR
VYRSNIPMVQFYITYQNNEGEKISKNLITKQISNNLTKYGSMHEPKKLNMPKPTISLN
NGEEFFEFKVPANKKLTFRLTSVIGSTTMYSCDVKMDYQLERNGNYELIRLKQIKDFV
NPALLTEPSQDEAYCKFVVKEIFEDGKETIIKSIS"

CDS 14335..14733
/codon_start=1
/transl_table=11
/product="hypothetical protein"
/translation="MYKLKLNPQTSGYGVTPGDDVKRQQMDGGRGRYYIDVKRNSHIV
DVNWNLSKTDFNKMMAFWRVYQNKPASFYADLVIDQGTRQQYLCNFIPNSFKTNEVNG
NLYRVNAQLEVVQNQPNLNADIALIKDWEV"

CDS 14733..15239
/codon_start=1
/transl_table=11
/product="DUF1833 family protein"
/translation="MDNEYAKFFFNRKVDVYQLECIELSHPSFMNTYRVVRNDDRGVY
VQHKEGSGQVYYEFLPVSIQRSGMLGDLDQTLTVSISGLGDVMPDEFERVIEGQYPDV

KPTVNYRIYSSDNLNSPMFYLLGLQLSSVAMNHKAVTFKAESPRLNTTKTGDIFALDR
FSGLKGAI"

CDS 15236..15598
/codon_start=1
/transl_table=11
/product="hypothetical protein"
/translation="MKSHDHLLDRQYDEDHYNCVHFVHEAAMDLYGIDRAEALELFMQ
PKGKITFLSSRLKLLNPLPMPKEGCIVAFHPRQRNKPPHVGLFRGQKILHLMESGVTY
LPEEVVMEMGFNRVSYYD"

CDS 15591..19016
/codon_start=1
/transl_table=11
/product="Phosphatidylinositol-specific phospholipase C"
/translation="MIKVIYKKDALSEEKTIEQAQTIGQWLTSKYEHMPEHVRIFHTT
SNMDHAEISFANEVTPKNAYDLKQLDFLPGTFIVVENPKWVAAIVSIVISIAIAFLMP
TPSIAQTTQNTNQSSSANNELSNRENKIRVNGRIADNYGAGWNTPDLIAVPYKVYENN
VEVEHVVGCIGRGHYKINGAYDGETNIVDIAGASVEVFRPGVDIVSGEPYFSLGTEIT
TPPLTVQHQTSVNGQVLRPADTQSLEGTNYLHFAYPNEILRATANNTDLTTKFVSNDR
VEITNASFTFNGQTFDLNGTYSVLSVADDRMTLSNPAAVNANWLKLKELNNQQTAALS
PKISSIGEKWIGPFILDNVERSRVLCNFVATNGLYTVSSGGYQAAVNVTIEVEVTPVN
ESGAAIGNPMLKQIILKGSAKSRQTVGATLDMVTFQGRCSVRARRLTPTPTVTTVVDE
VKWQALYGAYPLQSTVYEHETVFRARTYATTGALSVKSRKINFDLQRMLPTYKNGAMT
TELYPTSSFADALVSMALDDKIGRRSIDEIDLENIYRTYNDVVDYFGTPLAAEFCTTI
DDTNLSFEELVTNLCDAVFCTAYRQNNKLKLYFERPTDNSVMLFNFRNIIPDSYKHDL
TFGVMDDYDGLIYEYTDPTDDSRINIYLPDKGAKNPKEVKSVGVRNKWQAHFNAYRIW
NKMRFQRKSITFDAAPESELLVLRDRIAVADYRNGIHQSGEVVQQEGLVLTLSHDVDF
IAGKSYVIYLQMADGTVDLIPVTPGSAKNKVVLGRLPNGALKLSPDDFVNTIYTVVND
DTKGSLPYLVAKREPVDQFSNTITAINYDERYYLNDKDFIDVPVDDSPIYIRYDQLDI
NLARLYQMQRGDLPTTGEISFVVESGALVSSSSSYRPETRFVYKFDYNSSPPKQEFIA
PAATELPAIDTGEFPPDLVVNLTIKGAVVGRGGDGGLPHLAFGAWESDPDYNFTKTRR
DGFQGAPGLLNRHSKLNLIIDGGTLARGGSGGGATPSGIYTGLSYGVQGIPGGAGAPF
GRVMTGQPISSDSQDWRWYFGSYFNVLKITDAEASVPGKGYRTQNDRYGSPLSGDGGN
WGERGTKSTNDGTWNWKYHGTTEGQPGPGGPAIVGVAPLTTQLINGGKILQTL"

CDS 19084..19473
/codon_start=1
/transl_table=11
/product="Ammonia monooxygenase/methane monooxygenase"
/translation="MEPVSTSGFTALLKLYGIAIMVTLAVGLVAAVVLMTRMPRSPQE
WAVGLICTVVSSLAGGSFIIVKWGLHEWVTDVWGMIALGGFFFVCGLPGWALVRWIFN
FIDKQEGKTIVEVIKEFKKARKDIENS"

CDS 19473..20060

/codon_start=1

/transl_table=11

/product="Endolysin"

/translation="MPPSGGFLHLKETEMNIEQYLDELIKREGGYVNNPADRGGETKY

GITEAVARTNGFKGNMKDLPLDVAKAIYKKQYWTDPRFDQVNVISSLVAEELLDTGVN

CGTGFAKPLLQRALNLLNNQGKAGWPDLTVDGIYGPATLNALKTYLAKRGKDGEKVLV

RVLNIMQGQRYIEICERNPSQEQFFYGWIANRVVI"

CDS 20542..22860

/codon_start=1

/transl_table=11

/product="TonB-dependent receptor"

/translation="MKPLAALPLIFIAIQQVQAEERIAILKPLVFKANALKEKNYVVD

KEKVESSNTIGDALKNITGIQSTSFGPNAGAPVIRSLSGNRVGVIENGEFINGMNAFS

GNINIPFDPIFIEKVIVNKNTDNIRYGGNAIGGSVQIESGLIPKKIEEKPNKLDIVFR

KGFNDFDVKGFNFNINDQKNWSTNIRYSEYEISSYKIPGNSKAKLCEDQIFSNSGGIN

SALAASCQKDSRVQHIYNKSSQPYIDKFMTENPDWADGDFSFYTDKPTSIWGGKTYIN

PKNPEYIPNTPQNTIKKINTDVTPNYFKKLGNSYAQNENIGFGTTYFFDKGFIGLSAD

KKKSEYGVPGFSLQNQSFADSYETLPVGVKIDQNRFVLNSNFIQPISSAEEISLNFQQ

LSNKSGEYVGTAKANEYKIDNQLIELIMKQSSFKGLDGILGFSLKNRNIEGSGTQRYL

PNVSTISKAIFLQEELNIKQFTLNTGYRFERIEHELQDNDFKLARNASNSRIENRKYN

LNQYFIGGEYKVNNFINLKVDYGISERAPEVNELYSSNVHYSVMAQEEGDQNLKPEKS

KSLELGMFLNWNNWVMQLVGYQMDFENYMYLSHSGVAVQNRLPLKYWKQTDTDVKGFE

IDLIYDFNLAHIGNIKLGGLADFVKNKAVNPTDIRLANDGVYLPNMPTNRYGMFLEWR

NDSWKGKISSIYYDEPRYLGKNVIQEVPLSGYNLLEFKIDKKLKIKNASFDIFLNGTN

LLNEEARPQNSPLKYIAPLPGRAFQLGITMHI*"

CDS 23142..24527

/codon_start=1

/transl_table=11

/product="Phosphoethanolamine transferase EptA"

/translation="MALNVFKFKKICKDVTLLNFNLLLSIWLGLFLNIGFFKKIHQLT

PYNGIKSVLFLGATLVILIAAYNLIFQLINWKWTAKIFAILLIFIGGFSSYFVNTLGV

IISPDQIQNMVQTDVSEFTDLISLRFVLWTVFFVILPIFLITQVKFKQEKASRLLLKK

VFSLVASFAVVGVLLFTYYVDFAAIFREHRDLKGMISPQNSISSLMSYYHKKAPKKNL

PLVIYGQDAHQVQQVQKNLPKLMILVVGETARAESFSLNGYAKNTNPELSKQDIFNFS

QVSSCGTATAVSVPCMFSGMPRVDYDEQLASHREGLLDIAKRAGYQVTWIDNNSGCKG

ACDRVEQYQIPENLKKKWCKDGECYDDILIDSLKQYLATIAKDDDRPRLIVLHQVGSH

GPAYYKRAPEAYQPFKPTCDTNAIQGCSQTELLNSYDNTIVYTDHVLSQMILCTRQIS

QNPYPIRVLPS"

CDS complement(24489..24935)

```
                     /codon_start=1
                     /transl_table=11
                     /product="Transposase DDE"
                     /translation="MVGRVKLYISALQLENGELLLVVSPQFNANAIQDYALRWEIETL
                     FSCLKGRGFNLENTRLTDPRRVKKLIAVLAISFCWCYLTGEWQHDQKKAIKIKKHGRL
                     SMSLFRYGLDYVQMAIQRLIGFGKKEEFKEILAILRRQNPDRIRVL"
     CDS              complement(25010..25579)
                     /codon_start=1
                     /transl_table=11
                     /product="TnpA (transposase)"
                     /translation="MTHLNELYLILNKYLKWNKSHLKCFALIMLVIILKQTCNLSSAS
                     KALPIKCLPQSFYRRMQRFFAGQYFDYRQISQLIFNMFSFDQVQLTLDRTNWKWGKRN
                     INILMLAIVYRGIAIPILWTLLNKRGNSDTKERIALIQRFIAIFGKDRIVNVFADREF
                     IGEQWFTWLIEQDINFCIRVKKTSLSPII"
     CDS              25654..25977
                     /codon_start=1
                     /transl_table=11
                     /product="Phosphoethanolamine transferase EptA"
                     /translation="MINTLKEISKYQTGLWYLSDHGESTGEHGLYLHGSPYAIAPSQQ
                     THVPMIMWFSESWKQHNLAQVNCLSQQTKQKLSQDNLFPSLLSLLDVKTQVVNNKLDM
                     LSQCK"
     CDS              complement(26288..26776)
                     /codon_start=1
                     /transl_table=11
                     /product="SEL1-like repeat protein"
                     /translation="MKKIFLAALATIGLQACTTPYQEMGASGGVEATIIDDNVFQVRA
                     SVNGYTHKSIANQYALRKAAEVSKSLGCSYFSAINNTSQSYNQNISKVDAGLKTTPNG
                     VYYVSSAGTQYKLIKPSRNNTYVCFNEKPNTVLPGLIYNVKYVLGSPMPTGKFKVPNS
                     WR"
     CDS              complement(26866..28239)
                     /codon_start=1
                     /transl_table=11
                     /product="Recombinase"
                     /translation="MAQHIKFTKSVIDSIPLSEEKQIFYRDTVTIGFGLCVGKTKSYF
                     AEKKMPNGKSKRKVIGKHGVYTLEQARTEAKRLLILMDEGVDPVKQKRDLRASAIQND
                     ALQKLVPTLSEAYQYYKLRKKLAETSLIAYDGCIENYFSDWKDLKLDQITSAMIIDRH
                     LKLSKASPSRANLASKFLHALFNHTISRYKDESGNKILNIKNPVVIVKEEKAFNKIKR
                     RKGHVRADQREAWALAVATTYWMGEQNNDFRAYTNQDFLFLLALTGFRRSEAESVEWK
                     NVDLQFGTIKIINTKNHEDLLLPMGDTLWHIMRERKKRAGDNKYVFTDRNGVSHISDR
                     RAARDKVTENSGIEFTFHDLRRTFGTIANSLAIGSYTIKRLINHTTDDDDNDVTDGYI
```

QVSFDDLKKAMNMIEDVIISEPVKALIKSRLYFEKNESRNQAQELINHHIQVLDNFKM
AGQILDF"
CDS complement(28674..28937)
/codon_start=1
/transl_table=11
/product="DNA-binding protein"
/translation="MSRLTKLDRMTHAEKEAAKKEFWEAADNQTFPPETVAIVMHVSL
PWLQKKRCEGGGIPFSKPHKRQVNYMKSDVLAYIEQNKMAHTA"
CDS complement(28938..29660)
/codon_start=1
/transl_table=11
/product="CdiI immunity protein"
/translation="MSESTLWAVAMRPEGYSPFKQTPAASKEIAERAVERYRKMHEKE
GNNFFLEIFDDVIKVQKWHGSRKDHIKNLFYVESWFSEPMYQCFDLKTAERVFKFDEI
VICYKKGSAPLVTKSFDEAKLFYGSSETGFKYQIQPIEPPENLFNWFHPDIELFDTIE
EGAEAYTREQWAQLQMNLRVEIETQLLDYDEIPNIPEDAVVWPNWKPEPPEQGLFLIA
AFDSEDGPVLWWANPKAESKEK"
CDS complement(29657..30064)
/codon_start=1
/transl_table=11
/product="hypothetical protein"
/translation="MTGNERIPFESQFKTTEIFKRESAIRKNDILAFSETMNGYFNIV
TNDAWQLWNKAKAETVPDTPTPSVTLTCAELKEAFDFGAPDGEKDQFQMETEMTIKWL
QDGYDGEGYYCWYADLPEEGCIKLGVSESGAEG"
CDS complement(30065..30280)
/codon_start=1
/transl_table=11
/product="hypothetical protein"
/translation="MLGFMYYLQLLGYVIVRQGMDQAMFLTKHYAVPVAWRRITIDYH
NRLNKPAQQLYKEFVEWTKEEYAEMVA"
CDS complement(31247..32383)
/codon_start=1
/transl_table=11
/product="ATP-binding protein"
/translation="MRIRKMALPIITADQTLLVQAIIVYLYADPGLGKSSMGFTAEKA
ISFDFDRGAHRTGELRRGAVVQVQQWSDVANLTPQDLAPYKTVVIDTVGAMLECIKTH
LLLTANNRQKDGSLKLKAQGLANQTFKQYINTLISLGKDVVFIAHASEDQNGDQIIYR
PDLGGKNRNELYRIADLMGYLTTVTTGEGKNARVINFKPSPTHHAKNSGALGGETGEV
WVPDLKAHPTFLADLITQAKDHINTLTPAQLAAAKAQEELENWKQSCEEAEHAGDLNQ
LTESLDKEHMYYQNMRQAMLMRAKALNCTFDKQRGTWISPPEFNGISDQQRDELQNFI

AERGLDVKTVCEHFGIDALIQIEAAKLTAVKQEIETLAKTGMTA"

CDS complement(32380..32703)

/codon_start=1

/transl_table=11

/product="hypothetical protein"

/translation="MSNFKKHPDGYMSFLGRDDKGLYSVRIGWQVYASNANGSVLYKV

KDGVKTPLNVFRFQTSYPKVWNELTQEIDFQRRKQLAIKLRETNIPTYDRKAYKQKRG

FTGSR"

CDS complement(32696..32986)

/codon_start=1

/transl_table=11

/product="putative Ubiquitin-Binding Zinc Finger"

/translation="MKDYNCPTCKKMIPVDRSKIKAGDEVSFCRVTQSSKSARFSSRE

GIVNCREGDVVLVKYRKEIIPLNIRDVSPVDAPSPLTYAFVGTCECKEAEHV"

CDS complement(32986..33426)

/codon_start=1

/transl_table=11

/product="hypothetical protein"

/translation="MNVKTFSNKHKVTGVTAIAVLVALSSCEYRTANSSVPSNYSYES

EQVVASEYELLAVKKTGEKSGEAVIRIDGFKLNVSFDFDGVADSYGVAGSDFTTAEIT

NLAIDSVTDLSGKSFNDFTNHDDHKNINILLAGYIDRNNWLEAA"

CDS complement(33635..34138)

/codon_start=1

/transl_table=11

/product="Actinobacterial Holin-X"

/translation="MAKRYLPFYNNARFIALVLVGLFAIFSIIFKYLELNITINLVQF

SFVLLLPLSQIYLAYKGMLDALKLDGLNQSERDRLTSTVDIRSKSSLYVAMLFIILVF

SMYILNLLGLLSAKHLLALILSVGLTSIFSFFLAWSDLREISLLEKTLKDRKESREAK

AKVLSNK"

CDS complement(34140..35192)

/codon_start=1

/transl_table=11

/product="hypothetical protein"

/translation="MNPTQYFRAIREEEIMSKTVVKDKTVHYKKVDFLKGANLGQLLK

AQLLDKDSFYYKAINRQQFVSATKDDFILINHASSHQSMFFGELIIVESGKAQAVLKI

DSDDATEFPIKTYLTDDLPDDEDGVDATEVVRKEFIDSVLYFGVIDNHVAIIQSRSLT

ARTLESYLGWLLGEAAKALPENSALILKDAPNPTVKQKLESTPAKTISISSGIGSTEL

QPVHTVESSIPAKIDYKIEDNVVDVLKSAFGVDLENLKLEDGLDDANLKLKLTLTYNR

KTSKSGQKVIDTVASSMRHNDDYVITLEDGTKVTADNLKMSGKISVETINNKVYNDGL

KVQLYNWMTTNINFGD"

CDS complement(35199..35414)
/codon_start=1
/transl_table=11
/product="hypothetical protein"
/translation="MDNSKRPINQIIARINDAAKHGEALVLTAEEVKILSKDIGDKVF
IPVLTNEQVVQLVKEGKLGHKINNTKD"
CDS complement(35429..36193)
/codon_start=1
/transl_table=11
/product="LexA (CI repressor)"
/translation="MNTLAERLRYAMEVLPPKKIKGVELARAVGVKPPSVSDWLSGKS
KTMEGENLLRASKFLNVNPSWLASGTGEIQSSTRDKFKQLDIEAFKKKYNISDSDEAL
LFSTIIEKPFTPSSKRWVPVKAYSKMGMDGYFTDMGYEGNAGDGYVPTHSAGPRAYGI
KGTGDSMFPAIRNGWYVVCDPDADLVPNEFVQVCLKDGRCTIKEFVGINGGVLSLLSV
NGGERFFFEMDEVESITAITDIVPPSQHRQEHPYSH"
CDS 36302..36553
/codon_start=1
/transl_table=11
/product="Cro"
/translation="MNDAQLIDKLGGVTAVARLLGITPPSVSGWKAIPLDKKIRLAVI
AEDLGLTTRKELFPDNYQDIWIELRPQTTKSRNLGSLTA"
CDS 36564..36884
/codon_start=1
/transl_table=11
/product="Bacteriophage CII protein"
/translation="MSKVSNELPASASNNESLILQALNTSNQRQVAEKVGIDASTLSR
MKNDKKNNGLTEIEFISSLLTAIGLKVVPESDVYCSPEIAEATRVYLAHAFTSPEYMR
ILFK"
CDS 36841..37215
/codon_start=1
/transl_table=11
/product="hypothetical protein"
/translation="MHSLHLNTCGFYSNKKPLPAGTGVVRHSNEVDQMNTNNLSEQPI
ELNSPDFLIGDVVVLTKECRSFKSNDLFEVKNKTLTRLWTIKSENHLILVSSKEIRTA
TVAELNAKRRLTKAEQALAEVS"
CDS 37212..37511
/codon_start=1
/transl_table=11
/product="Iron-dependent Transcriptional regulator"
/translation="MNSQFKYKPEYKQTQEIQSFFDPALVILNELHDRNRKNLRAKGY

DENNAAITREEFSQTMAQRFRINQWLAGQIVNSLANADLVQKFGGYVKPKVGVHE"

CDS 37504..38385

/codon_start=1

/transl_table=11

/product="Bacteriophage replication protein O"

/translation="MSNFVPNSFQVPNAFVDEVLNKISDAACKIYLVICRKTRGWNKE
MDSISLTQFEEITGKSRPTVVKCLNELIKVGLVVEQPSTIHGNTFKLGNDTSVGLVLK
FPSKNFLLPEIYGQTSKNSLPLLVKNFNYTSKNFLPLLVKIFNTQSITIKNNSQSNKK
INKKREPVSEKPKSEKPTEFNPRLVELPACVDPELWNNFVDMRISIKKPLSENAVKLI
LKKLISFGPLANQSLENSIIGNYQGVFEPRQNQIQENPQSHNVPEEPGYFTQMYAESN
RSNVIDVTPVSQDFGGY"

CDS 38388..39188

/codon_start=1

/transl_table=11

/product="Replication protein P"

/translation="MNELAPFESFLKELIAAYRTKYAVQFNKNFPVEGKNAVPMQIVE
QQLAKALVGVTPNQLQRGLALFYASTNTYMPNFAEFRAMCMGDDWWSAEKAWVKACEY
TQISQHKKVTLPDGREQNQEITTLTKFVLDQVYSLIQDGEMYKAKMEFIKIYDEYKAE
AQLKGKTQAWYQEPILLAQKNEQKVHKPVSNDEAQKHLQSLMERLKINGRKPVPVQKL
KAKEKEPELKQELGPDPFDNPHEYAEMCRREGMPIPRNILKLIDGANI"

CDS 39185..39319

/codon_start=1

/transl_table=11

/product="putative phage replication protein"

/translation="MNKFEILAWGLLISFFTAAISGAVVWWWLARKELDEKGASHESN
"

CDS 39288..39728

/codon_start=1

/transl_table=11

/product="Ribosomal protein L7/L12 C-terminal domain"

/translation="MRKEPAMKATKLIRDKGLQYAKEIVDSAPDNATEWNEGYEFQCG
QSVEISPADREKYFVDLVELKRLVESLKIINDLGGVEKLTPAFITTDKHVGYTHVRMV
GNGRLSFLDDFCDFIPDGSISIKRVMTAIRDHESIYGGGESHAN"

CDS 39718..40140

/codon_start=1

/transl_table=11

/product="hypothetical protein"

/translation="MPTRYNTGEYSYDLEYHYGDMSASMEMLRARLIELLTPHLSGRY
VKWREAYFTWFTKCGGDSGWMFCVGPHEFHIDGALRRYYSGSIDITYNQKDRYFLVGE
KKKVKCKACKGFGFIRDDGWGHIDKCEMCDAEKGASHE"

CDS 40133..40357
/codon_start=1
/transl_table=11
/product="carbohydrate binding domain-containing protein"
/translation="MSEFEGKSGKWAWEIQKEQQAKVEELQKRLDGALKETQYALQYV
EEDMRGNHEFLQMAMIRTLKAIEQVLKGGA"
CDS 40357..40758
/codon_start=1
/transl_table=11
/product="DUF559 domain-containing protein"
/translation="MSSVSIAEYRKLFPIKKNKKRRSAKQVARQPSVGEVVLATHLRA
CKIGFEQEYKFHPERKWRADFLIKGSKILIEVEGGIWSGGRHTRGKGYLGDMEKYNSA
AMMGFTVLRFSTEQVKSGMALKQIELLIKGK"
CDS 40769..41521
/codon_start=1
/transl_table=11
/product="hypothetical protein"
/translation="MLVEKFDFIELLRLAIAQGKAEGKKISKDVVLGELALLSPAAKL
WATVLIEKVDFERIAIITPAQKQTETFYSKYDFNFQTERRIEDIPGKVEFVRGEIKSG
NFFRARNKLAVEIHKEMVKKKFTPTNAQGDLTNLAKGMAEIILRGHVFVKAMCGVCQG
LGKIETFGLNGFPNGARFCEKCNGTGKRPYTLKEKMNIAGIDATKTAYIKSYQKFELF
GESIVAEWENEIRTRISRSFRFELPDSQETYA"
CDS 41943..42398
/codon_start=1
/transl_table=11
/product="hypothetical protein"
/translation="MTQYLMFAENIYNKIKDEELFSHDCIENMNLLMTCIRREIKGTE
FKLKYNFIDFVELFSRPLDECKVKIDVSLIPPHNSEGEYILWLAGLIEKITEGGPKPP
PPIKKFIPEYMSLKFELDFLPLNEEKIQNEGKEITDYFNSKLYKATFKK"
CDS 42460..42894
/codon_start=1
/transl_table=11
/product="hypothetical protein"
/translation="MDSTEYFWLTRKKEPKTKPKSRPLPKAKQKYLEAEATLKEELED
LAIGFESKFQPIHTKHWRFDFHIVKLRLLIEIEGGPWSGGRGGKLSNKAWSLNRYDHA
EEMGYKIERFHPDSILSGYVINWIKSELARIEDGADQTISTD"
CDS 42863..43504
/codon_start=1
/transl_table=11
/product="hypothetical protein"

/translation="MEQIRPFPPTDFIDQAEEEEAIRLTPAPDLKKWVVANYLTIGGP
IYNPDHDHIAELLHDNDEFLAFAWASSAYKSKQAMVLGQCEKVMFNVGGWRKARQEQQ
MRDWFGFVPTYLITVDASFCERANDTEFCYLLEHELYHIGVMRDEDGEIVYSDSSGLP
KHYLAGHDVEEFIGVVKRYGPSKNVKRLIEVAKNPPFVSNLDISKCCGNCVIN"

CDS 43563..44033

/codon_start=1

/transl_table=11

/product="DUF2280 domain-containing protein"

/translation="MATLKEPVKIFIVQSLACRDTPQEVAELVKQEFGVDIDRVQVAT
YDPTKVAGKNLSKKYVELFEKTRDEFDKGLIDIPIANKYYRLKQYQRQLEKTRNVKTA
LKILEQAAKDIGGQFTNRQEITGKDGGPVQTVNSEIPVPMEDYLKARREVLDEY"

CDS 44023..45450

/codon_start=1

/transl_table=11

/product="Terminase large subunit"

/translation="MSTDAARDKAIRIEAQEDLYFFTRYMFKERRGYKWMQNWHHLEI
CEALMKVYRGEIKRLIINVPPRYSKTEIAVINFMAWCFGKNPDCEFIHISYSAMLAAN
NAFQIRTLVQEEAYRKVFPELTLRDDSKAKDFWRTSQGGVCYATGTGGTITGFGAGKL
RKGFGGCIIIDDPHKAHEASSKTIREGVIDWFQNTLESRTNSPDTPIIVIMQRLHEDD
LAGWLLGDRKDGVPVAGGNGEVWEHLCLSAIQEDGSALWPAKHNIQKLRLMEQAAPYV
FAGQYRQMPSPPAGGFFKPDNIQIVDALPADVVKQVRAWDFGATENEGDFTVGVREAL
GADGFTYIVDVTRGQLGPDNVNKRLEQTAKIDGKKVSVRLPQDPGQAGKSQASSFVKL
LAGYSVIAKPISGDKLTRAQPFAAQVNVGNVRMLKGEWNKDFIDELRHFPNGTHDDQV
DAASDAFNELHEGFEAFFADMGFAR"

CDS 45447..46898

/codon_start=1

/transl_table=11

/product="DUF4055 domain-containing protein"

/translation="MSDVTFQHAEYVKNLPYWQKLDDVCEGEDAVKAKGEKYLPMPNA
HDKSPANKSAYEAYLTRAVFYEVTGTTLNSLVGAAFATDPSFKFPPELAHLERNANGA
GLSTYQLAQNGIRHLLKHYRCALYVDYPDVPPARNLAEFKAQKAYPMIHLLNALDVVN
WDSVMIDNQKKLCLVVIREFKSERGADGFSKTEQEQYRVLRLEQEGNGEYIYSVQVYT
KGEKGNWVGGEKKFPTDYNGNFWTYIPFTFVGAIDNSEEIKKPPLLPLANLNLAHYRD
SADFQESVFYMGQPQYYAKGVNWEWYDQAKKRGIYIGAKVLLPLPENGGLGIVQADPN
TLAREAMKDKWEKMKEMGARLIEKGSGSKKTATEANSDDAVQHSVLSLCVVNMNEALS
AALRWAAKFVTPNVDVLTKDDLMFEISQEFNKQGYLAELARQLFEAALQGRSSFKSWW
EYNQTGMFPKQKYEEELQNVEAEQDGTLNQKVE"

CDS 46900..48003

/codon_start=1

/transl_table=11

/product="Minor capsid protein"
/translation="MATDIKKLFEVLTQHQAYLYRASSKTVNELLALFNDDTSKMLSK
LRDLLDELNESEKVALAGGKYTTSNLREIRDLIAQWFASVNLALPEAFAVSATALAVY
EANYVAKLYGAKINKPDGEKLFLSAKKVPLAGGALVDDLLSRIAESARQKVEYAIRDG
INSGKTNQEIVQRIRGTKRLNYEDGILNGTKTDIERTVRTVRSHVANQAYLNSFNQIG
FEYVRFVSVLDGRTSKLCASLDGSVWEINDPAKRVPPLHPNCRSILVPVEKDGQLVGE
RPFVMDERRVKDIPKEERSQLIGQLDANTTFKEFFKKTDDFFQREWLGPKRFKLYKDG
KFDFDKFFDPEGRFYSLDDLRKLDEKAFKKLGL"

CDS 48012..48440
/codon_start=1
/transl_table=11
/product="hypothetical protein"
/translation="MLYFLKHQNLYNMKTIAFVCLTLISITCLAEPSQKYLKEYDRLS
EALESAMANAYSFDPATGQVKQATQGLEAKNNLCRAAQAKLNLTTFLKDNLEESKELY
KSIDGAETLDKNYLSGQQQEQQNLVSNLKKDLVGTGFNCE"

CDS 48539..48781
/codon_start=1
/transl_table=11
/product="hypothetical protein"
/translation="MSESKVRHLVLKRVSDKSSHLALCDEETGIPLAGLTAVKMNCSV
FEGPATITATFDVGGPQGIRLVGDEPRQKVWSAKET"

CDS 48999..49190
/codon_start=1
/transl_table=11
/product="Occludin homology domain"
/translation="MPSPIIQYFQYEHLPEHLQQVSKPIGDLARQMDEQLPDGPEKST
GLRKLLEAKDAFVRQALSK"

CDS 49304..50071
/codon_start=1
/transl_table=11
/product="Telomere regulation protein Stn1"
/translation="MKLKTVTIDGKVYAEVDGDKPIYIHDDGKEMPHDAPHSVATIAR
LNNEAKTHREAKEAAEKALKAFEGIEDPAAAKKALQTIQNLDDKKLVDAGEVEKVKAE
AIKAVEEKYAPIVAQRDALEASLHKELIGGGFARSKYIQDNIAVPVDMVQATFGHHFK
IEEGKVVAYDPNGEKIYSRVRPGELANVDEALESLVGGYQHKDLILKGGKGTGGGFQG
GGKGGAPTGMKRSEMSVSQKADYIKEHGNDAFLKLPN"

CDS complement(50449..50619)
/codon_start=1
/transl_table=11
/product="hypothetical protein"

/translation="MRPNLSPNFRIPRVNALRPSTTILPFATMSGLLEIAAFKEAKLY
SNRPATNASARS"

BASE COUNT   16086 a   9005 c 10650 g 15188 t

ORIGIN

```
        1 tcgaaaggtg aaaatcctga ccttacatta cttggttcaa gcgctaactg gatcaaatat
       61 gcaaccagca acaaaatgac agcaggtacc ttacttgatt tatcgggtac agcgacaact
      121 ggttaaaacc taaaaattaa aacctaaggg ggctaataag ccctcttttt tattattaag
      181 agaaaagcgc catgaagatt atctatacac gcattgcagc agcggctgca ttagagacag
      241 gcattattgc taaccctgac tattatgaaa acccaaattt gaaagcaaaa gaggtaatta
      301 tttacggtaa ttatccaaag attcaaaagg attatgaatc tttggaagtt ccagttgaag
      361 ttcgtaagtt ggaagtgcca caaaaaacga ctttggccac agtaaatgtc gcagtgggaa
      421 ttacccctga acttcaagct gtgatggatg atgcaaaagc tgaatgtgaa aaggtagttg
      481 aagaaaacac tcagcttaag cagaaaattg ccatcttaga gcaggccggt ggtaaccagt
      541 cagagttgtt atctgaaaat tcacgattaa aagatgcagc agtcttagca gataaagctc
      601 tcaaagatgc tgaagctcaa gtggtcggta taaaaactga atttgaagct tttaaaaatg
      661 atattcctgc aatgcaggca cgtattgctg aattggaagc tggaaaagcg gcagaaaacc
      721 cagctacaga aacggcagct aatgattttg aaaactggtc aaatgatcaa ttaaaagagt
      781 atttggctag taaaaacatt ggttacaagc cgtctgcaac aaaagcagaa cttcttaaat
      841 taatcccgaa ggaataatgc aatgagcttt attactgtag atgacgcaaa ttcaattttg
      901 ggcagcgatt ttgcaccaga cagtgataag gctcgtctgg tgaagctggc aaatgtgtgg
      961 atgaaaaaca gaattggttt tgtaccagat ccaattgatc cacttcttaa ggacgcggct
     1021 tgtgaaatta tcaaaggaat tctggccaaa gtaatttata acggcaaaga gcagcagttg
     1081 aagcgtaaga aagttaaggc tgattctgtt gagtcagaaa aagaatttca agacggatct
     1141 gaagcaatct ctagctttga acagatagca attgatttta ttgattcact tgatttgaaa
     1201 gatccaaatg caagttttaa tggctttggc ataccacttt acagggcatg atatgggctt
     1261 acgtgacgaa attcaggcag acattgctga agcatttaat gatgatttag cagatgccat
     1321 tcataccttt acatgtgagc ggatctctaa aacgaattgg gatcctaaaa ctgaaactta
     1381 tgttgaagtt aaagaaaact attctggccg tggcgttctg tttggctcat acagtcaata
     1441 tgagatccaa acacttggag tactggccac ggataaaaag gcaaccgtgc ttcaaaatga
     1501 agtaactatg actccaaaaa ttgaagatga atggttaact gccttaggct cattccgggt
     1561 aattaatatt caacaggatc cagctaatac tatttggaaa tgtcagcttc gaaaagtgta
     1621 ggggctaaaa tggttaatcc tgattatgtt cctgaatggt atatctcgcc ttttcaacat
     1681 gtgcagtaca cgcttgctcg aaatcaacta cacatggatt tgttatttga agatatggat
     1741 aaggccgatc aatttttgga tatgggagcg gatgcgcaag ttagtacttt ttctgatggt
     1801 gcatatgcaa tcgtccaaat tggtgatacg gcggataaag accgaattca agtttatgga
     1861 ttgcttttac atgaagctgt tcatgtctgg caaaagatta aaaagctcat gggtgaacga
     1921 gaaccgagct ctgagtttga agcttattca attcaggcga tcgctcaaga ccttttttaaa
     1981 atgtatgaag aaagcgaggt gaatgatggg atggaagggg aaaaagccaa ctgaatttag
     2041 ttttgatgtg tctaaagcag cagaagacca ggtgaagcat attgttatgg atactgtgca
     2101 atccttagtt aatttaagtc ctgttgatac tggagcatac cgtgcttcac atattgtttc
```

2161 ggttggatct gctgatttcg gcgtgcgtga acctgaaaca aaccctatta acgacgcagc

2221 gattcaggca atgaagatta agttaggtaa tttggtttat atccagaaca ataaagctta

2281 tggaccgcgc ttagaaaacg gctggtctga tcaagcacca caaggtattt atggcctcac

2341 gtttaacttt atttctcaaa agtacggtgg ctaaaatggc aatgacttta gagcagacaa

2401 ggcaagctat tattgatcgc atgcaaagct ttacgggtat tgctcaagaa cggattcagt

2461 atccaaatgc acctgacttt actgttccaa caaaaggtgt atggtgccgt ttaacgattg

2521 caggtggccc gagttttacc tcaggcattg cagataagcc atgtacacgc cgtaccggta

2581 atatcatgat ccaatgcttt gaccgattac atactggaga gaaggccgta acggttctaa

2641 gcgatgcatt gctggctcat tttgaatatt tcacaatcga acacttagaa tgtttgaatg

2701 gccaatctat ttatgcgggt aaagatgctg atttcattca gtataatgtg agcattgggt

2761 acaaggtgaa ttgatatgtc atgtatgctg actttagaag aaatcgaaat taaacggcaa

2821 gaactggaac ggcatcttga agatgttatg tctgttgagt tgagcaaatg gcaatctgaa

2881 aacaagctat gtgtttctga tgtgaatata cgcttggcta atgttgttag tctcggaggg

2941 cctaaacata acgttgttac tggagtaagt gtcgatttag ataatgagct ttgagttcaa

3001 gaaaaagcta ctgcaaggcg attattttta atgacctcag catattatca tttgtgatta

3061 cattctgtta cagtaatgga aatttataac aaatggtaaa acatgaaaaa atcaacttta

3121 ggctggggtg ccgcaggatt agtagcttta gggatttttg gttcaggcaa tgataactct

3181 ccaaaacaaa cttcagactc agaaaatgcg cagagtgcag tagaggaagt tatcgaatca

3241 aaatatatca acactaattc tttaaatatt agagataaac caaacggtca agtagtagga

3301 aagttaggac gtggggaaaa agttgatatt tatgagatga aaggaaactg ggcacgtatt

3361 tccttaaatt cctcatcacc tcagtggtta tcaacaaagc tattatgtga aacggatggc

3421 tgttttaaac aaaagtctcg atcaaccacg tcaaataatt atcaggcctt aaaatctcat

3481 cctcatcatt ctgaaagaaa acagaaaaaa acctactacg atagtgattg ttcatgtgct

3541 gtggtggatt attgcgtggg tcctagaggt gggcactact gtattacgag tggaggaaac

3601 aagagatata aacctagata ttaattaatt tgaattatga gacctccatt ttgagaggta

3661 ctttatgtct taatcactac cacctcatcg gtggtttttt tatgtctata ggaatcactt

3721 atgagcaatt ttgtatttaa gcgtggtgac actttcaact taaatcttca gctagttgat

3781 atggatgaag ccctgcaata tccaccagat gatgtgcgcc gtgcaattga tcttacaggt

3841 tataccttca cttcacaggt taaagctctg gctgatggtg ctactgtggc cacattaact

3901 tgtactgcat taagccaaag cacacagaaa gggtggctga acattaaatc tagtgcaagc

3961 actgcaactt ggcctttagg gctgtgtcag atggatatta aagctgtagt tagtggcact

4021 acacagcaca ctgaaacttt gactttccaa gtgattgacg gagtaacagc ataatggcaa

4081 atcttgtttt taaatttagt tgggatcatc ggccattccc atataacgcc tcacagggca

4141 agcggcagtt tatgttgcca tttgcgtcag gtattcccaa tctggcaccc aacttttctc

4201 aagtagttgg tactgcagct atctctcaag gtggaacggg ggcaactaca gcggctggtg

4261 cacgagctaa cctaggcgca gctgcaagtg gggtaaatag tgatattagt gagcttaagg

4321 gacttacaac ccctttatca atttctcagg gaggattagg agctgataat gcacagacag

4381 ctagaatgaa tttggggtta ggaactgctg ctgtactagc gtcaacaaca agtcaatatg

4441 atcctacgcc gggacgagca ctaagagtcg gtgattgggg gataggggct gaaggttctc

4501 gtgtatctga tatggttgct cctcttaata atggtttttt tcgaacagat gacactttaa

4561 caaatgatac tggtaatagt attggtcctt atggtttctt tttacactgt acccgacgct
4621 caatgggttt atatacaaat ggaagtcatt catttcagct tgggaaagct gcctcatatt
4681 ctgccctgaa gtatcgattt aataatagtg gtacttggtc taattggttt aatttattga
4741 ctgcacaaaa tactacaact gatggaaatg gttttattaa agccgcttca ccagtcgtta
4801 agcttttcca aagtcatatt gagctaaata acgatgctgc caagcaaccg atcacttttg
4861 acaaattagg cactggtgac tatctgatta agggctcttt aggctttgca caggaaggtt
4921 ggtatatcga agtacctaag gatgccaacg gtaatacggt agtagcagtt gaatattcaa
4981 ccttagaaaa tggtgatctt tctattaaaa cttataaacg taagtttgat gtggaaaagg
5041 cagccattgt agctgatctc gaaaatccac ttgatattcc agaaggccgc tggattgata
5101 tccgtctgca tgaagaacct gaaccagagc ctgaagagcc gttgagtgaa acaccagtgg
5161 atttccagcc gactaactta tctcaggcag ttgctgcagc catgaatggc gtggaaccgc
5221 cagaaatctc agaaacagac gaaacacttt aataacccgc ttaaacagcg ggttttttat
5281 tgcctaaatt ttggagaacc ataaatgagt tcaggcgcaa aaattcgatt atatgcttgt
5341 gaggaagcag ttttaggaac aactcctgca aatccagtct ggtacaccgt tcgccgtgtt
5401 actgatagtt tgactgaaaa tgttactact gaagatagca gtgaagtagt tgattcacgt
5461 tttcgccaag gcgctgttgt aacggaagcc gaagtaactg gtcaactaga gtttgaatta
5521 tcactaggta cctttgactt attcttaaat gttctcgctt tcaataactg ggctgcaaat
5581 gctttaagtt ttggtggtgg agtacgtaag tctcttacct tggtaaaagt ctttgaagat
5641 attggtcaag tctttattta tcgtggtatt caagtgaata caggtgaaat gacgatccag
5701 accacaggca aaatcactgg taactttggt ttagtaggta gctcatttac gcgacagcag
5761 gttaatcctg ttacaaatcc tattccagca tcgactcgcc ctctggtgag tatgccaaat
5821 gttgaaaagc tacttattaa tggtcaatca attcagggta aagcttgtct gcagacactt
5881 accatcaact ttagtaataa tctggaagcg atccgttgta tcggttctgg taagtacacg
5941 cctgagtttt acttagagaa aatgatggat attggcgtaa atgctaattt catgttttca
6001 gcaacatctg cttcttggat tgatgctatt aaaacccgtg atgtatttac attgaccttc
6061 gatattacag ataccaaagg cagtaagtac tcgtttaact tcccgcaact tgaagttaag
6121 gaagcaaatc accctgatgg tggtggcgat gacattatta caatagatat caattttgcc
6181 caagtgcgta ccagtccaac gattgtacgt gctcttgtgt aatcaactta ttcagtaaca
6241 aagcctatgg aaacccatgg gctttttat ttctaaaaat tagaggttgt tatggcttta
6301 aaagtcggaa ttattaaaag ctcagacgta tcaaaatggt gtgaatacaa gggtgctgat
6361 ggcgatgtac aggctgagtt caaagtccgt ggtatcgctt ataagccttt tcaggtagct
6421 attgaacggg caggaaacca gatctcgtct aaaggctatg atgtgatggt caaagatgaa
6481 gatgccaagc tttaccacga gcttttaatg gatgcatgcg cggcccactt aatcgaagac
6541 tggaaaggtg tggtatttgc cgaaatcgta gacggtaaaa ctgttgagtc cgaaaagcca
6601 tatacacctg agaatgcctc aaagcttctt aatcttggtg atattggtat ttcaatctgg
6661 ctattcatta aagaacaggc ccagaagatt caggaagacg cagacaagga caaggcttta
6721 attctgggaa agtcatggag ctctacaaat accaaaaaac gtatgcgtcg aaaacgccgc
6781 acgaaatcga gcaaatcaag ttcttaggcg gccgtattcc ggatccgcca gaatattcgt
6841 atgcggctga ttcaattctt tcggcattta gcactatatg tcgatccaga cgttatgagc
6901 aaagcatacc gttatcttta gatcagcagg ctatcaatgt ctatgctgag cataatgatt

6961 tgccagtggc tgctcatatt tttaatgact gtatttttgc gttggataat ttgtttttgg
7021 aggagtgcca taagaagata tcaaccaaaa gcaaaggtaa gtgaccaaat taggtattgc
7081 cagggactga aaagcctaat ttggtcaaaa cgtcaaacaa ttaagcagtt gctcttaaac
7141 gcgactcaaa ataacgcagt cgatgttaca aaatacttga tctggattga cagaaaatta
7201 cctttaaggt gttgcgcgtg attatcaaat gatgaataat caccttaccg tcaatatttg
7261 acggttcagc attcttttac tctttccaag aaccttggtg tttgcttgta tgtgtttaac
7321 attaactgaa gctaaacaaa aacttagagc atttgctaga gatactagca aaatcaagtt
7381 aactgcacat gcaaaagaaa gaatgaaaga acgctgtatc tctatgaagc aaattatttg
7441 ctgttttgag catggagata ttactgaggg gccgtaccca aatactcgtg gtgattgcca
7501 gttaaatgtt tctgttcgca ctgcaggcga atacataaca acagctgttg caatcaagca
7561 gagcgagaac ggtgaattct cagtagtagt cactacattt agagagtagg ctaaattatg
7621 tatcactatg aagaatgcgg tctgagcaat atttggctgc gcaatggatt tacaattgaa
7681 aatgatgaag actatggtga actcgtatct attgaatctg ttcatgagct tcataatgcc
7741 attgggttgt tcttaattac gcaaaagcct gacttgaatg gtgaggaaat tcgtttttta
7801 cgtaaagaac taaacttgtc acagaagaat cttgctgggc ttttaggagt cagtgagact
7861 agtattagac attgggaagc tgatcgcggt ttaattggta aacctactga gctattactt
7921 cgtgcattat ataaagagca tgttcaaggt gatggcaaac taagaagtat gattgagtca
7981 ttaaatcatc aggaacgaac tttagtacca agtgaaatta gtttttcata tggaaataac
8041 cattcatggc atcaaaccaa ttgtgaaata gcttagttag ttttatttga tagaaaccac
8101 cttcgggtgg ttttccttta tgtgacattt agtaaccagt ttgttaaagt tagtacactt
8161 tataacaaac ggtgaaattc atgaaaaaaa tattggctgc gggtttaatt ggtcttgggt
8221 tggtggggtg cgctactcca gcctataatt atcaagctat acctaaaaat ataagcaaac
8281 cgccaattgg atcagttaat aaagcatttg taggggatca aatgcttgaa cagggaatgg
8341 tggttgatcg tgaagttcta aacgtccctg aaaatattaa aattagtttt gcttattcac
8401 ttacttcagg catttactta aaaacaggca aaaatgaaaa agggcaatat tttcagccat
8461 tcaacactgt cagtggtggg ggatggttcc agaaaaaccc tttagctgac ccatttaaag
8521 tagttatgtt agatactgaa ggtaagctct gtgtagtaac agtatttaat gcaaaaaact
8581 gtactgataa acatcaagct actatgaaga cagtagcaat tgcatcagat aattccttcc
8641 aacaaacatt aatttatagt ggaaaatttg gaaataaaat taatgtcggg taccgtgaat
8701 tctcaagtaa tcaagcacgt cctgcattca ataatgatgt tgaatatgat ttaagccaat
8761 ctaagcaaat aggttataaa ggtgctttat tggaagtaat tgatgccact aatcaagata
8821 ttacttacaa agttttgaag aactttaaca aggtagatta agatgagtgc accacaatat
8881 aaaccaatga gagaaagtga agtttgtaat gctatcgggt gggtgttaat agctcttggc
8941 tttatcgcag gtttttttatt tattcttgca tttggtcgaa ttgaagtagc ttcttactat
9001 ggtaaagaaa cggtttggtc tggagttatg atagcaacag gaatcggaat tatatttaat
9061 ggattccttg caggctactt atttcaaaaa gtagctagta ttcttcgtta ccatgagaat
9121 aaataatatc ttgcataagc accctaggat gctttttaaa attggtttaa ctaccctgct
9181 tggtaattat atttaactta aaaagaacta cccactcatt gagtgggttt tttattgcct
9241 agaggaaagt aaaatggcac aagaatcccg tttggtcatt gttattgatt cgcaaaatgc
9301 tgaacgtaat gcgcgtaatc taggcaatga acttgttagc attgaacgta aaggtgaatt

9361 tgcatctaag tctatggaca gcttgtctgt agccaccaga gctttagctg gacacatggc
9421 tggtttatta acagtaggtt cagccatttc aaagatggat acatatactg gattacaaaa
9481 tcgccttaag ttagtcacta acaatcaagt tgaactaaat aaagcaacgg aagacacttt
9541 ccgaattgct caaaaaacct attcagcttg ggattctgtg ttacaggttt accagcgttt
9601 tagtgataat gccaaaactt taaacctcac aatggatgac acagcacgtt taactgaaac
9661 agtttctaaa gctgtagcaa ttagtggtgc aagcgcagaa gctgctgatg cagctttagt
9721 tcagttcggg caggccttgg ctagtggaac gttgcgtgga gaagaactta attctgtaat
9781 ggagcaaacc ccagcactag caaaggctat tgctaaaggt atgggtatta ctgtaggtga
9841 attacgttca gtagcagctg aaggaaaaat tacttcacaa gaaattgtaa aagcgcttag
9901 aaatgtagaa tctgatgttg atgctctttt tgctaaaaca gatatcacaa tcgggcagtc
9961 tctcacactc ctaaacaacg agatcacaaa atttgttggc gaagcaggta agggaagtgg
10021 tgcggcacag gtattagctg gatcagttca aactcttgca agtaatttag atttaattgc
10081 tgatggggct ttagtagttg gtattggata tatcactcgt gcaattttga tgaagagcgc
10141 tgctattaaa gagggaatgg cttcaacttt agcgagccgc caagcatctg tattaaatgc
10201 tcaagcagaa tatgcagaag ctaccgctgc tttgaatgca gcaaaagctc atctcgcgaa
10261 tgtgcgagca acaaatgcag aaacccaagc taaatttggc gcaacagcgg cagcaactcg
10321 atacgcacaa gcacaggcag cagtaactgc tgctacaaat gcacaaacag cagctcaaat
10381 taagctaaat actgcaactt caattgcagg gagactagct aaaggggcgt ttggattaat
10441 tggtgggtgg gctggagttg caacattagg agtaatggga ttagcggcag cctattctta
10501 ttttaataat aaggcagagg aggcaaagca aaagcttgct gaacaagcta aagttgctga
10561 gaaagctgat gaggagttaa aaaaattaac tggcaatgat aaggctaaag cagttaatga
10621 tttaactact gcttttaatg cacaaaataa agcattagag aaatcatcgc gtgctgtagg
10681 gtctgcatta attgatatcg agaactatgc acgaggaaat agggaggttg aaaaaatttc
10741 ccaagaagcg agaactggaa ctatcagcta tacagaagcc attgaacgtc taaataaaat
10801 taagttgcct acagatctat atgaaaatct gaaaaaacag gctgcgcagt atgatgacaa
10861 tgcatctaaa gcaagtttat cagctgagaa acttaaatta ttaagagttg aagtgaaact
10921 tggaggtaat gaagcacaaa atgcggcaat tcagcatcaa aaacaagcgg atgctttagg
10981 aaatactgct actgaagcag aaaaggcaac taaggctttg caagattatc aagccaagca
11041 aaaagatagc gttattgatt caatctataa atcaggttgg cttgataaag gttacactgt
11101 tgctcaagct aatgccattt tagaactgca aaaagctaaa ggaatgagtg caattttgtc
11161 taaagatgaa attgatagcg cacttagaaa tctcaagatc atcgaagaac aacaggagcg
11221 agaagataaa ttaactgaag ctaaaagaaa gcagacgcag gaaattgaaa aacaagcaaa
11281 acttactaaa cgcttggtcg gtatttccgg tcaatccggt attggtactg gtccacatct
11341 tgacgtccga tatggtggct cattgtcagg tcagaaagtt tctaatgaac atctggctcg
11401 attacaggcg ggaggaaaac ctttaacttc ctacaagatc agttctaatt atggtccacg
11461 aaaagcccca actaaagggg cttcttcatt tcataaaggt attgattttt caatgcctga
11521 aggaacacca atcacgacca atgttgctgt gaaagatatc aagacatggt atgacagcaa
11581 gggaggtggt tatgtcagtg aagtgatctt tgaggatgga gtgtctctta agcttctaca
11641 tcaatctccc aagatgcaga gcaaggtgaa aggtggtgca agtaaaggaa gtgataaagc
11701 agctggtgat attcaatctc aacttgaacg tcaacaggat ttgcaacggt cacttgaaaa

11761 tgaggtggct agtgaagtcg gacggattaa caataataga aaggcaagac tggaggatgt
11821 tgataaagca aactttagcc cggaacgtac tgcagaaata aaggcggaaa taaatcgtcg
11881 tgcagataat gatattgcta tagccaaaca agcccttaga acgaaattgg aagactataa
11941 ggagttccag aaaaccgagg aacagttact agaagagtcc tttaaccgta aaaagttcaa
12001 tgcagctcat gaccttgaat taagtaagtt tgagcagaag caagctgttg aattgctgga
12061 acagcaaaaa cagcaagagt tagggttatt aaaactagct caggaacagc ggttgtttca
12121 agcccgttta tctctgcttt ctgaaacgca agccatgcag gaacgttaca gactcgaacg
12181 ggaggaaatt cttaagaata ccaagctttc tatagaagag cggcaaaagc taatcgcatt
12241 atctaaagcc aatcaggata aagagacacg cgataaagtg aataatgctg ttcaaaactg
12301 gggtggtatc caagcggata tgaatggtac cggagaattt ttcagacagg atcaggaacg
12361 atttagccgt ttaaatgctg caaatgattt agcagatagt caatttgctg ctactgatct
12421 tgatgaaaaa aatggtttag atgttctaaa tgcacacatg gaagcaggac tcatcaagca
12481 acaggacttc gaaaaccgga aaacagctat cattcaagct gctcaggacc aacgcaatca
12541 gatcgctgcc gaatatgctc agaatgctca ggatattgaa gataagtatc accaagatcg
12601 attgaatgct caaattgctc ttggtggcca aatgatgggt tcactcacat cgatgtttgg
12661 ttcaatgttt ggcgagcaat caaaagcata caagatcatg tttgctgctg ataaagctta
12721 tgccattgca gctgcaggta tttctattca gcaaagtatt gcaaaggcgg ctagtgttgg
12781 ttttccagca aatatcccat taattgcaag tgctattgca caaggtgcaa gcatcattgc
12841 aaacatccgg gcaattaaag atcaaggctt tgctgacggt ggttacactg gatcaggtgg
12901 gaaatatcag cctgctggta ttgtccataa aggagaggtg gtctggtccc aagaagatat
12961 taaacgctgg ggcggtgttg gcttagtcga gaaaatgcgt aagagtgcaa accctgaagc
13021 ttttctcaat aacaatgcct tggcagatag tgtcatgcgc cgtgcaatga tgagctctag
13081 tgcctttata gaaagccaaa agcaggctga catctttaat caaccggttc aagatactca
13141 gattatctat aagggtaata gagacacacc taaattagct tcttctggaa atttagactt
13201 attccatgat ggcaaggtct acttctcatc caatggttta gttcaggatc gttcaaatct
13261 ggatgatgtt caggacttta ctttaggaag tacttcacgc cctcaagctg agatgatgcc
13321 ttcaattgag ccagcttcac cgacaatcaa tttcaaaatt gaagtgatta atcaggtgag
13381 tggggcgaca gttgaagccg aacaactgga cgagcaaaca gtccggatca ttgttaaaga
13441 tgaactggat aagcagcttc caagaacggt acctaagctt gtaagtgatc aaatcgcaaa
13501 tccaaactca accattagtc ggtctttgac tgagaatacg acagcgagaa gaaatcgtac
13561 ttaataattt gaaccctttt cggagggttc attttcataa tatttaaatt tcaaggtgat
13621 agagtctgtt ggcattaaaa ttgatggtta agacatgaaa aaaataattg taatttctac
13681 aacactttta ggccttacgg gatgtgccat tcctgcggta aataatctcg taagatccac
13741 aaatatgtat caggatgatg tttcgggaaa tactgcaaat ttaagggttt atagaagtaa
13801 tattcccatg gtgcagttct atattactta tcaaaataat gagggtgaaa aaatttcaaa
13861 aaacctaata actaagcaga tttcaaataa tttaacaaag tatggctcta tgcatgagcc
13921 caaaaaatta aatatgccta aacccacaat cagtttaaat aatggtgaag agtttttga
13981 gtttaaagta cccgcaaata agaagttaac tttcaggctt acttctgtta ttgggtcaac
14041 tactatgtat agttgtgatg taaaaatgga ctatcagttg gaaagaaatg gaaattatga
14101 attgatccgt ttaaaacaga tcaaagattt tgtgaatcca gctttactga ctgaaccatc

14161 tcaagatgaa gcctactgca agtttgtagt aaaagagatt tttgaagatg gtaaagaaac
14221 tattattaaa tcgatttctt aatgttaaat cgtttttgta attaatttaa atatctaaac
14281 cttatttcat caaaccacct ttcggggtgg ttttttatta cctgaaggaa agttatgtac
14341 aagttaaagc taaatcctca gaccagcggc tatggcgtaa caccgggtga tgatgtgaaa
14401 cgtcagcaga tggatggcgg tcgtggtcgc tattacatcg atgtgaaacg taacagccac
14461 attgttgatg tgaactggaa cttaagtaaa accgatttca ataaaatgat ggctttctgg
14521 cgggtctacc agaacaaacc agcctcattt tatgcggatt tggtgattga tcagggaaca
14581 cgtcagcaat atctatgcaa tttcattcca aactcgttca agaccaatga agtcaacggc
14641 aacttatatc gtgtgaacgc gcagctcgaa gttgttcaaa accagcctaa cctgaatgcc
14701 gatatagcat taattaaaga ttgggaggtc taatggataa cgaatatgcc aagttctttt
14761 tcaatcggaa agttgatgtc tatcaactgg agtgtattga gctatcacat ccttctttta
14821 tgaatactta tcgggtagtc cgtaatgatg accgaggtgt ctatgtacaa cataaggaag
14881 gatccggtca ggtctattat gagttcttgc cagtctctat acaaagatcc ggaatgcttg
14941 gtgatctgga ccagacatta accgtttcta tctctggtct aggtgatgtg atgcctgatg
15001 agtttgaacg ggtaatcgaa gggcaatatc cagatgtaaa gccaaccgta aattaccgga
15061 tttacagttc agacaatctg aactctccaa tgttttattt acttggactg caactctcaa
15121 gtgtcgccat gaaccataaa gctgtgacat tcaaggctga atcaccacga ttaaatacca
15181 ctaaaactgg ggacattttt gcactggatc gctttagtgg tttgaagggg gctatatgaa
15241 aagtcatgat catttgctcg ataggcaata tgacgaggat cactacaact gtgttcactt
15301 tgttcatgaa gctgcaatgg acctatatgg catagatcgg gctgaagcgc ttgaactctt
15361 tatgcagcct aagggcaaaa ttactttttt atcttcacgg ttaaaacttt taaatccgct
15421 acccatgccc aaggaaggct gcatagtcgc cttccatccg agacaaagaa ataagccccc
15481 gcatgtgggg ctttttcgtg ggcaaaagat tcttcacctc atggaaagcg gagtcactta
15541 tttgcctgaa gaggttgtga tggaaatggg gtttaatcgg gtcagttatt atgattaaag
15601 ttatttataa aaaagacgct ttgtctgaag aaaagacaat tgagcaggct caaaccattg
15661 ggcaatggct cacttcaaaa tatgaacata tgcctgagca tgtccgtatc tttcatacta
15721 caagcaatat ggatcatgcc gaaatttcat ttgcgaatga agtcacacca aagaatgcat
15781 atgacttaaa gcagcttgat ttcttaccgg gcacttttat cgtagttgag aaccctaaat
15841 gggtcgcggc tattgtttcg attgtgatta gtattgcgat cgcattttta atgccaacgc
15901 catcaatagc acaaacgact caaaatacta accagtcttc ttcagcaaac aatgaacttt
15961 ctaaccggga aaacaagatc cgggtgaatg gtcgtattgc tgataactat ggagctgggt
16021 ggaatactcc cgacctaatc gcagtacctt acaaggtata tgaaaacaac gttgaagttg
16081 agcatgtagt gggctgtatt gggcgtggac actataaaat caatggagct tatgacggtg
16141 aaaccaatat tgtcgatatt gctggcgcat cggtagaagt ctttcgacca ggtgtagata
16201 ttgtttcagg tgagccatat ttctcgcttg gtaccgaaat taccacgccg ccactaacgg
16261 ttcagcatca aacttctgtt aatggccaag ttttacgtcc tgctgataca caatctttag
16321 aaggtacgaa ctaccttcat tttgcatatc caaacgagat tcttcgggca acggcaaaca
16381 acacagattt aaccactaag tttgtaagta atgaccgcgt agaaatcacc aatgcctcat
16441 tcacgtttaa tggccagact tttgatttaa atggtactta tagcgttcta tcggtagctg
16501 atgaccgtat gacgttatca aatccggcgg ccgttaatgc taactggtta aagcttaaag

16561 agttaaataa ccaacaaact gcagctttgt caccaaagat cagttcaata ggtgaaaaat

16621 ggattggtcc attcattctg gacaatgttg aacgtagccg ggtgctgtgt aattttgtgg

16681 ccaccaatgg actttatacc gtttcttcag gtgggtatca ggccgctgtt aatgtcacga

16741 ttgaagttga agtaacaccg gtaaatgaat ctggtgcagc gattggtaat ccgatgctga

16801 agcagatcat tttgaaaggt tcggcaaagt cacgtcagac cgttggtgca acacttgata

16861 tggtcacgtt tcaggggcgt tgtagtgtcc gtgcacgccg tttaactccg actccgacag

16921 tcacaacagt tgttgatgaa gtaaagtggc aggcgcttta cggtgcttat cctttacaaa

16981 gcacagtgta tgaacatgaa acggtttttc gtgcgcgtac ttatgcaacc actggagctt

17041 tatctgttaa gtcccgcaag atcaattttg atcttcagcg aatgttgccg acttataaaa

17101 acggggcaat gacaacagag ctatatccaa cgtctagctt tgctgatgct ttggtatcta

17161 tggcactcga tgacaagatt ggccgccgtt cgatcgatga gattgatctt gaaaacatct

17221 atcggaccta taatgatgta gttgattatt ttggtacgcc gctagcggct gagttctgta

17281 ctaccattga tgatacgaat ctatcttttg aagagctggt taccaatctt tgtgatgcgg

17341 tgttttgtac tgcatatcgg caaaacaata agctcaagct ttattttgaa cggccaactg

17401 ataactcggt aatgctgttt aacttcagga atatcattcc ggatagttac aagcatgacc

17461 tgacctttgg cgtgatggat gactacgacg gactgatcta tgaatacacg gatccgaccg

17521 acgatagccg tatcaatatc tatttaccgg ataaaggagc caaaaaccca aaagaggtga

17581 aatctgttgg tgtacgaaac aagtggcaag cgcatttcaa tgcataccgg atttggaaca

17641 agatgcgctt ccagcgcaaa tccattacct ttgatgcggc accagaatca gaattactgg

17701 ttttacgtga ccggattgct gtagcggatt atcgcaatgg tattcatcaa agcggtgagg

17761 tggtacagca agaaggttta gtcctcacct taagccatga tgtagatttc attgcaggca

17821 agagctatgt gatttatctg caaatggcgg atggcacagt ggacctgatt cctgttacac

17881 cgggttcagc caaaaaataag gtggttttag ggcggttgcc gaacggggcc ttaaagctaa

17941 gtcctgatga ttttgtaaat accatctata cagtagttaa tgatgatact aaaggttcat

18001 tgccttatct ggttgcaaaa agagaaccgg ttgaccagtt ctctaatact attacagcaa

18061 ttaattacga tgagcgctat tacctcaacg ataaagactt tattgacgtg ccggttgatg

18121 attctccaat ttacattcga tatgaccagc tggatattaa tctggcgcgt ttatatcaga

18181 tgcaaagagg ggatttgcca acgactggcg aaatcagttt tgtagttgaa tctggtgcac

18241 tagtttctag ttcgagttct tatcgaccgg aaaccagatt tgtctataaa ttcgactaca

18301 actccagtcc gccgaaacag gaatttattg cccctgcagc gactgaacta cccgccattg

18361 atactggtga gttcccacct gatctggtgg taaatctgac tattaaaggt gctgttgttg

18421 ggcgtggtgg tgatggcggt ttacctcatt tggcatttgg cgcatgggag tccgatccgg

18481 attacaactt tactaaaacc cgacgtgatg ggtttcaggg agcacccggt ttattgaacc

18541 ggcacagcaa actaaacctg attattgatg gcggcactct agctcgaggt ggatctggtg

18601 gtggagcaac accaagcggt atttacactg ggttgtctta tggtgttcaa ggtattccgg

18661 gtggagctgg tgcaccattt ggacgggtca tgacaggcca gccaatttca agcgactcac

18721 aagattggcg ctggtatttt ggaagttact tcaatgtctt aaaaattact gatgccgaag

18781 cttcggtacc cggaaaaggc taccgaaccc aaaatgaccg ttatggatcc ccattatcag

18841 gtgatggcgg aaactggggc gaacgtggta ccaagtctac taatgatgga acatggaact

18901 ggaaatacca tggcacaact gaaggtcagc cggggccagg tggacctgca attgtgggag

18961 ttgcaccact gacaactcaa ttgattaatg gagggaaaat cttacaaacc ctttaaacct
19021 tataagaact ttgagcaccc aattcgggtg ctttttttatt acctaaattt tctggagaaa
19081 ttaatggaac cagtttccac tagcggtttt acagcacttt taaaattata tgggattgca
19141 atcatggtga ctttagcagt cggtttggtt gcagcagttg tattaatgac tcgtatgcca
19201 cgctcaccac aagagtgggc agttggtttg atctgtacgg ttgtatcaag tttggctggc
19261 ggctcattca ttattgtgaa gtggggactt catgaatggg ttactgatgt atgggggatg
19321 attgctctag gtgggttctt ctttgtttgt ggtttacccg gttgggcttt agtccgttgg
19381 atttttaatt ttatagataa acaggaaggt aaaacgatcg ttgaagtgat caaagagttt
19441 aagaaagcca gaaaagacat tgaaaacagc taatgccgcc ttcgggcggt tttttacatc
19501 tgaaggaaac cgaaatgaac attgaacaat atctggacga gttaattaag cgtgagggcg
19561 ggtacgtaaa caacccagca gatcgaggcg gtgaaacaaa gtacggtatt actgaagcag
19621 tagcacgtac taacggcttt aagggcaaca tgaaagattt accgcttgat gtggccaaag
19681 ccatttataa aaagcagtat tggacagatc cgcgatttga tcaagtgaat gtaattagct
19741 cgttagttgc tgaagagctt ttagatactg ggtaaattg cggtaccgga tttgcaaaac
19801 cactcttaca gcgtgcttta aatttgctga ataaccaagg taaagcaggt tggccagatt
19861 taacagttga cggaatttat ggtccagcaa ctcttaatgc actcaaaact tatctggcca
19921 agcgtggaaa agacggcgaa aaagtcctgg tgcgtgttct taatatcatg caagggcaac
19981 gttacattga aatctgtgaa cgcaatccta gccaggaaca gtttttctat ggttggatcg
20041 ccaatcgagt tgttatatga aagtctttca ttgcagacga tcaaagatag ctttaacaat
20101 tacattgctg tgcattctat tttcaggatg cacagctcat acgatcaata gcaatgtcaa
20161 tgtctcaatt tgcgctagag cattataaaa aaaagccctg aatgatcagg gctatttaat
20221 aaacttcaat tcacatactt ctacaaagct cgagtagtct tatgtttgtt attgtatagc
20281 ttgataatcc tctatctttc atcctagggt tgtgcacata cttctatcct taaattgtgt
20341 aatgaaattt cttctgttgt agttgagaag cttttcaatc tgttaaaaga atccaaaaag
20401 catcaaatga ataaagaaac tacttattta acttgaatca tcattttctg tttactaata
20461 aatgaaagtg cttggatgta tagggattgt ttttttaatg ttatgttata atataacctt
20521 ttcttaaaaa aggggggat tatgaaacct ttggcagctt taccgttaat ttttatagca
20581 attcagcaag tgcaagctga ggagagaatt gcaattttaa agcctcttgt ttttaaggca
20641 aatgcattaa aggaaaaaaa ttatgtggtg gacaaagaaa aagtagaaag ttccaataca
20701 ataggtgatg ctcttaaaaa tataacaggt atacaaagta cttcatttgg acccaatgca
20761 ggtgcaccag ttatacgtag tttgagtgga aatcgggttg gagtaattga aatggagaa
20821 tttattaatg gaatgaatgc atttagtgga aatattaata taccattcga tccaattttt
20881 atagaaaaag tgatagtgaa caaaaaataca gataatattc gttatggcgg aaatgcaata
20941 ggcgggagtg ttcaaataga atcgggttta attcctaaaa aaattgaaga aaagccaaac
21001 aaattagata ttgtctttcg gaaaggattt aatgattttg atgtaaaggg gttcaatttt
21061 aacatcaatg atcaaaaaaa ctggtctaca aatataagat attcagaata tgaaatttct
21121 tcttataaaa ttccaggaaa tagtaaagcc aaattatgtg aagaccagat tttttcaaat
21181 tcaggtggga tcaatagtgc attagcagct tcctgtcaaa aagatagtag agtccaacat
21241 atttataaca agtcgtcaca accatacata gataaattta tgactgagaa tcctgattgg
21301 gcagatggcg atttttcttt ttatacagat aagcccacat ctatatgggg aggaaaaaca

21361 tatataaatc caaaaaatcc agagtacata cctaatactc cgcaaaatac aataaaaaag

21421 atcaatacgg atgttacacc aaattatttt aaaaaattag gtaacagtta tgctcagaat

21481 gagaatattg ggtttggaac aacatacttt tttgataaag gtttcattgg attgagtgca

21541 gataagaaaa aaagtgaata tggtgtacca ggattttcat tacaaaatca atcttttgct

21601 gattcttatg aaacattacc ggttggtgtg aaaatagatc aaaatcgttt tgtattaaat

21661 tccaatttta tccaaccaat ttcctctgca gaagagataa gtttgaattt tcaacagctt

21721 tctaataaat ctggagaata tgttggtact gcaaaagcta atgagtataa aattgataat

21781 caattaattg aattaataat gaaacagtca tcctttaagg gattagatgg gatacttggt

21841 tttagcctta aaaataggaa tatcgaagga agtggtactc agcgctattt acctaatgta

21901 agtactatta gtaaagctat attcttacaa gaagaattaa atatcaaaca atttacttta

21961 aatacgggct atcgttttga aagaattgaa catgagcttc aagataatga ttttaaacta

22021 gcaagaaatg ctagtaattc aagaatagaa aatagaaaat ataatttaaa ccaatatttt

22081 atcggaggag aatataaggt taataatttt ataaatttaa aagttgatta tggtatttca

22141 gaaagagcac ctgaagtgaa tgagctctat tcaagtaatg ttcactattc tgttatggca

22201 caagaagaag gcgaccagaa tctcaaacca gaaaaatcta aaagtttaga gttaggtatg

22261 tttttgaatt ggaataattg ggtcatgcaa cttgttgggt atcaaatgga ttttgaaaat

22321 tatatgtatt tatctcattc aggtgttgct gtgcaaaatc gattaccttt aaaatactgg

22381 aaacagacag acactgacgt caaagggttt gaaattgatt taatttatga ttttaatcta

22441 gctcatattg ggaatattaa actaggtggt ttagctgatt ttgttaaaaa taaggctgtt

22501 aatcctacag atataaggtt agctaatgat ggagtttacc taccaaatat gcctactaat

22561 cgttatggca tgtttcttga gtggagaaat gattcttgga aaggtaaaat ttcaagtatt

22621 tactacgatg agccaagata tctaggtaag aatgtaatac aggaagttcc tttatctggt

22681 tacaacttac ttgaatttaa gatagataaa aaattaaaga ttaaaaatgc ttcatttgat

22741 atatttttaa atggtacaaa tctgctgaac gaagaggcta gaccacaaaa ctctcctcta

22801 aaatatattg cccctctacc agggcgagca tttcaactgg ggataactat gcatatttag

22861 gttgctaaag atataaagtt agttcttatc tcctgtcttc aaatttagta ataaaaatat

22921 agtcttaaag ccgaataatt aatcggtttt aagattttaa aataatcaat tataaagttt

22981 aaaatttttt atttataaaa tgtgttcaaa taatgaactg attttttagc tcaaggagcc

23041 atcgtcatat aaataaagaa tattacaaag gttctttttt aatttttctt taaggtatgg

23101 tatctaatct tagcattttt tatatttaat ggtttttaaa aatggcactt aatgttttta

23161 aattcaaaaa aatatgcaag gatgttacac tattaaattt taatttgctt ttatctatct

23221 ggctaggttt atttctgaat ataggttttt ttaaaaaaat ccatcaactt acaccttata

23281 atggtattaa gtcagttctt ttcttagggg cgacattagt tattttaata gcggcatata

23341 atttaatttt tcaattaata aattggaaat ggactgccaa aatctttgca attttattga

23401 tatttattgg tggctttagc tcttattttg taaacacatt gggtgtcatt atttcacccg

23461 accaaattca aaatatggtg cagaccgatg tttcggaatt taccgatcta atctctttac

23521 gctttgtttt atggacagtt ttttttgtta ttttgcccat ttttttaatt actcaagtta

23581 aatttaaaca agaaaaagca tcacggttgt tattgaagaa agtattctca ctggtagctt

23641 catttgcagt ggtcggtgtt ttactttttta cttactatgt cgatttcgct gcaatatttc

23701 gtgagcatcg tgatttaaaa gggatgattt caccgcaaaa tagtatttca tcgcttatgt

23761 cttactatca taagaaggct ccgaagaaaa atctgcctct tgtgatatat ggacaagatg

23821 ctcatcaagt tcagcaagta caaaagaacc tccctaagtt aatgatactt gttgtaggtg

23881 aaacggcacg tgccgaaagt ttctctctaa atgggtatgc aaaaaatacg aatccggagc

23941 tttctaaaca agatattttc aacttttcgc aagtgagctc atgcggtacg gcgacagctg

24001 tttctgtgcc atgtatgttc tcgggtatgc cacgtgtaga ttatgatgag caattagcta

24061 gtcaccgcga aggtttatta gatattgcaa aacgtgcggg ttaccaagtg acttggattg

24121 ataataactc gggttgtaaa ggtgcatgtg atcgcgttga acaataccag attccagaaa

24181 acttaaagaa aaaatggtgt aaagatggcg aatgttatga tgacattctc attgacagct

24241 taaagcagta tttggctact attgccaaag atgatgatcg tccgcgtttg attgttctgc

24301 atcaagtggg tagtcatgga cctgcatatt acaagcgtgc acctgaggca tatcaacctt

24361 ttaaaccgac ttgtgatact aatgcgatac agggctgttc gcaaactgaa ttgctaaata

24421 gttatgataa tacaattgta tatacagacc atgtattaag ccaaatgatt ctctgtacac

24481 gacaaatttc acagaaccct tatcctatca gggttctgcc ttcttaaaat tgccaaaatt

24541 tccttaaact cttctttttt cccaaaacca attaaacgct gaatcgccat ttgaacatag

24601 tctaaaccat agcgaaataa actcattgag agtcgtccat gcttctttat ttttatcgct

24661 tttttttgat catgttgcca ttcacccgtt aagtaacacc aacagaagct tatagctaac

24721 accgcaatca atttttcac tcgtctaggg tctgtcaagc gcgtattttc aagattaaac

24781 ccgcgtcctt tgagacaact gaataaggtt tcaatttccc agcgtaatgc ataatcctga

24841 atagcattgg cattaaactg aggagaaacg acgagtaaaa gctctccatt ttctaactgt

24901 agtgcactta tatatagttt cacccgacca accaaaatcc gtcgtttacg acattcaatt

24961 tgaccaactt taagatggcg aaataaatca ctaattttat gattctttcc taaatgattg

25021 gtgacaatga agttttttta acacgaatgc agaagttgat gtcttgttca attaaccatg

25081 taaaccactg ctcaccgata aactctctgt ctgcgaacac attcacaata cggtctttac

25141 caaaaatggc tataaagcgt tgaatcaaag caatacgctc tttcgtatct gaatttccac

25201 gtttattaag caatgtccaa aggataggta tcgctattcc acgataaacg attgcgagca

25261 tcaggatatt aatatttcgt tttccccatt tccaattggt tctatctaaa gtcagttgca

25321 cttggtcgaa tgaaaacata ttgaaaatca actgagaaat ttgacgataa tcaaaatact

25381 gacctgcaaa gaagcgctgc atacgtcgat aaaatgattg tggtaagcac ttgatgggca

25441 aggctttaga tgcagaagaa agattacatg tttgctttaa aataatcaca agcatgatga

25501 gcgcaaagca ctttaaatgt gacttgttcc attttagata tttgtttaag ataagatata

25561 actcattgag atgtgtcata gtattcgtcg ttagaaaaca attattatga cattatttca

25621 atgagttatc tatttttatc gtgtacagag caaatgatta atactctaaa agaaatatca

25681 aagtatcaga caggtttatg gtatttatct gatcatggcg aatcaaccgg agaacatggt

25741 ttatatttac atggttcacc ttatgcaatc gcaccgagcc aacaaacaca tgtaccaatg

25801 attatgtggt tctctgaaag ttggaaacaa cataatcttg ctcaagtgaa ttgtttaagc

25861 caacaaacta aacaaaagtt aagtcaggat aatttattcc caagtttgtt aagtttgctg

25921 gatgtaaaaa ctcaggtagt aaataacaaa cttgatatgt tgagccaatg taaataaata

25981 tatttttaat ttatataaaa cgaagcaaaa tagacttgct tcgttttata aaatgtattg

26041 gttcttttat atttaaaaaa taagagggca gtcgtaaccc ggataaacac cattcttata

26101 gtcgaatgtc ggttcgaata gatctagtag gtgaactcga tctttattta taggttcata

26161 gttcgcgcac atacaaagta acctctttat gattttcgaa attacttttc catcataact
26221 aatataaata aaaagagggt aaataataga ctgtagacgc tattattcac cctcatggta
26281 tatatcttta acgccagcta ttcggaactt tgaatttacc tgtaggcatt ggagaaccca
26341 gaacatactt aacgttgtaa attaaaccag gcaaaacagt gtttggcttt tcattaaagc
26401 aaacataggt attgtttctg gatggtttaa ttaacttata ttgtgtacca gcacttgaga
26461 cataatagac accatttggg gtagtcttaa ggccagcatc aactttagaa atattttgat
26521 tgtaactctg gcttgtattg ttaatagctg agaaataact acaccctagc gatttagata
26581 cttcagcagc tttacgtaat gcgtactggt ttgctatcga tttatgggta tatccgttta
26641 cagatgcgcg tacttgaaag acattatcat caataatagt ggcttcaacg ccaccagaag
26701 cccccatttc ttgataaggt gtagtacagg cttgtaaacc aattgttgct aaagcagcta
26761 aaaatatctt tttcataatt tatataaagt gataaatgcg tcacattaat tttaactatt
26821 tagaagaatt tttcaattac ttaaggattt cttaaaactt gagttctaaa aatctagaat
26881 ttggcctgcc attttaaagt tatctaacac ttgaatgtgg tggttaatta attcttgagc
26941 ctgatttctc gattcatttt tctcgaaata taagcgactt ttgattaatg cttttactgg
27001 ttcagaaata atcacgtctt caatcatatt catagctttc tttaaatcat cgaaagaaac
27061 ttgaatatat ccatcagtca catcgttatc atcatcgtca gtggtatggt taattagcct
27121 tttaatcgta tagcttccta tggccaagct gttcgcgata gtgccaaagg tgcggcgtaa
27181 atcatggaac gtaaattcga taccagaatt ctcagttact ttgtcacgtg ctgctcggcg
27241 gtctgaaata tgggaaacac catttctatc ggtaaagaca tatttgttat caccggcacg
27301 tttttttcgt tcacgcataa tgtgccaaag ggtatcaccc ataggtagca aaagatcctc
27361 atggtttttc gtattaataa ttttgatggt accaaattga agatctacat ttttccattc
27421 aacagattct gcttcactac gtctaaaacc agttagagca agtaagaata aaaagtcttg
27481 gtttgtgtac gctctaaaat cgttattttg ttcacccatc caataagtag tggccacagc
27541 caatgcccat gcttcgcgct gatctgcacg aacatggcct tttctacgtt taattttatt
27601 gaaagctttt tcttctttaa cgataacaac cgggtttttt atatttagaa ttttattccc
27661 ggactcatcc ttatacctgc taatcgtatg gttaaagaga gcatgtaaaa attttgatgc
27721 aagattagct cgggaagggc ttgctttcga aagcttcaga tgacgatcaa taatcattgc
27781 actggtgatt tggtcaagtt ttaaatcttt ccaatcacta aagtagtttt ctatgcatcc
27841 gtcataggca attaaagatg tttccgccag ctttttacgc aatttatagt attggtaagc
27901 ttcactaagg gtagggacta gcttttgtaa ggcatcattt tgaattgctg aagctcgtaa
27961 atcacgcttt tgcttaactg gatctacacc ttcatccatc aagataagaa gccgtttagc
28021 ttcagttctg gcttgttcta atgtatagac gccatgtttt ccaataactt tacgtttaga
28081 cttgccatta ggcattttct tttcagcaaa atagctttta gttttgccca cacataagcc
28141 aaatcctata gttactgtat ctctgtaaaa gatttgtttc tcttcagaca aaggaataga
28201 gtctattacc gatttagtaa atttaatgtg ttgagccatc tttaaattaa gcaatacaaa
28261 gcaataccta acgatacaat acaataattg tgaagtctat taagagtcta ttttgaaggt
28321 ttggtctatt aaaaaagaga gttttaagta ggggattttg ttttaagtta ttgttatcta
28381 ttaaaaataa atgctccgaa gatgccgctg catgtcgtta cccttgaacc ctaaagttca
28441 gcgggtcttt ctcaattcta gcaatacatt gcaatattaa gcaataccta gcgatattaa
28501 aaaatcaata tttttaataa tttatattaa agcaatacaa tgcaatatta cacaatcttt

28561 agcaatacaa aaatagtcta ttaatggtct attttgataa atacggtcta tttttcaagt

28621 ttaagtctat taaaggtcta tttttggtga ttaaaaaagc ggcacttagc cgcttatgct

28681 gtatgtgcca ttttgttttg ttcaatataa gccaaaacat cagacttcat ataatttact

28741 tgtcgtttat gaggtttaga gaatggaatg ccgccacctt cacatctttt cttctgcaac

28801 cacggtaagg atacgtgcat aacaatagct actgtttcag gtggaaaagt ttgattatca

28861 gcagcttccc aaaattcctt cttagcagcc tctttttctg catgagtcat acgatctaat

28921 ttagttaaac gtgacattta tttctcctta ctttccgctt taggatttgc ccaccaaagt

28981 acagggccat cttctgaatc aaatgctgca attaaaaaga gtccttgttc tggcggttct

29041 ggcttccagt ttggccaaac tactgcatct tccggtatat ttggtatttc atcgtaatct

29101 aatagttgag tttcaatttc aactctaaga ttcatttgaa gttgtgccca ctgttctctt

29161 gtataggctt cagctccttc ttcaatggtg tcaaacaatt caatatctgg atgaaaccaa

29221 ttgaaaaggt tttcaggtgg ttctattggc tggatctgat atttaaaacc cgtctcacta

29281 gatccataaa atagttttgc ttcatcaaag cttttggtta caagaggggc agagcctttc

29341 ttgtagcaaa ttactatttc atcaaattta aaaacacgtt cagctgtctt caaatcaaag

29401 cattggtaca taggttcact aaaccaactc tcaacataaa atagattttt aatatgatct

29461 ttgcgggaac cgtgccattt ctgaactttg ataacatcat caaaaatttc taagaaaaag

29521 ttgttgcctt ccttttcatg catttttcta taacgctcaa cagctcgctc agctatctct

29581 tttgaagctg ctggcgtttg cttaaaaggg ctgtaacctt caggtcgcat tgcaaccgcc

29641 cataaagttg attcactcat ccttcagctc ccgattcgct aacacccaac ttaatgcaac

29701 cttcctcagg taaatcagca taccaacagt agtatccttc accgtcataa ccatcttgga

29761 gccatttgat ggtcatttca gtttccatct ggaattgatc tttttcccca tctggcgcac

29821 caaaatcaaa ggcttctttt agttcagcgc aagttagagt gacactaggg gtgggagtat

29881 ctggcaccgt ctcggctttg gctttattcc ataactgcca agcatcatta gttacaatat

29941 tgaaatagcc attcattgtt tcactgaatg ctaggatgtc atttttacga atagcacttt

30001 cacgtttaaa aatttctgta gttttgaatt gtgattcaaa agggatacgt tcattacctg

30061 tcatttaagc caccatctct gcatattctt ctttagtcca ttcaacaaac tctttataaa

30121 gctgctgcgc gggtttattt aaccggttgt gatagtcgat cgttatgcgg cgccaagcga

30181 ctggtaccgc ataatgcttg gttagaaaca tcgcttgatc catgccttgc cggactatta

30241 cgtagcccag caattgcaag tagtacataa aaccaagcat gtgtttttgg ctcactttct

30301 tgtactgatc tttcatatta gaaaccgtct cctaataaat aatcaggctc agcctcttga

30361 agtggcgtag atgtagggtt ctctaattca aagcggcgtt tcttaacaaa gtccatgagt

30421 cgtgattgaa tctgtggatc tcgtgcggcc acatctattt ccaaagcatc taatgttgtg

30481 agatcgggcg cgttttggat ctggaccatt agtgaaggtg gttcagttgc ttgcgcctta

30541 gatttttcga gctcttcaag acgtttgtga gttgcaagta gcaaaggctc catttgttta

30601 tcagaccaag tgcgtgtata tcgataaact gcatttactt cgtctggtgt ttttgagtcc

30661 ttaactcgtt gcagcagggt atcaagtttc ttctgatatt ctgaatcagc ggtttctgat

30721 tcaatagccg gctcagctgt ttgtgctttt tctacaacct ctggttttga atttttaact

30781 tttagagatt ccgcatgact ttctgaaggc ttttcttctt cgacttcttc agtaggctta

30841 tttagaagtt ttagaatgtc ttccgcaaac tcaccaccgc tgattttaat aatcgcgcag

30901 caatgagcaa atgcattatc aaaacttgag tgaacttggc catgctggag catgcgcaat

30961 tgtcctttag atccattcca cttaaactgc tgcacaccta attcaacagt tgggctaggg
31021 taagagcaag tagaaccttt ttctggcgca actcttaatg gttctggtac ctcaaattca
31081 ccaataaaaa tagttctagg ctttaattga aattcgaatt tatcaaaaac atcaaagcca
31141 aagtcataag ggttaaatgg ttcccagcca ttacgctcag tattatttac taaaagtaat
31201 tcaccgttgg cccaagcaag tttggcttca actttattta gaattttcat gctgtcatcc
31261 ccgttttcgc taaggtttca atttcttgtt taactgcagt tagttttgcc gcttcaattt
31321 ggatcagggc atctatgccg aagtgctcac aaactgtttt tacatcgagg ccacgttcag
31381 caataaagtt ttgaagttca tctctttgtt gatctgagat accgttaaat tctggtggac
31441 taatccaagt gccacgttgt ttatcaaacg tgcaattcaa tgctttagct ctcattaaca
31501 ttgcttggcg catgttctgg taatacatgt gttctttatc aagcgactca gttaattgat
31561 taaggtcacc tgcatgctca gcttcttcac agctttgttt ccagttttct agctcttctt
31621 gggctttagc tgctgcaagt tgtgcaggcg ttaaggtgtt aatgtgatct ttagcttgag
31681 taatcaggtc agccaagaaa gtaggatgtg ctttaagatc tggtacccat acttcaccag
31741 tttcaccacc taaagcacct gagtttttcg catgatgtgt aggcgagggt ttaaaattaa
31801 taacgcgggc atttttacct tcacctgtag taacagttgt tagataaccc atcaaatctg
31861 cgatacggta aagctcgtta cggtttttac cacctagatc tgggcggtaa ataatttgat
31921 caccgttttg atcttctgat gcgtgtgcaa tgaaaacaac atctttacct aaactgatca
31981 aagtattgat gtattgcttg aacgtttggt tcgctaaacc ttgagccttt aactttaaag
32041 agccatcttt ttgacggtta ttagcagtta gcaatagatg ggttttaatg cattcaagca
32101 ttgcacccac ggtatcaatg actacggttt tatatggtgc taagtcctgc ggagtaaggt
32161 ttgcaacatc actccattgt tgaacctgta caaccgcacc acgacgtaat tcaccagtac
32221 ggtgagcacc acggtcaaag tcaaaagaaa ttgctttttc cgcagtaaag cccatcgatg
32281 atttacctaa acccggatca gcgtataggt acacaataat tgcttgaacc aataaagttt
32341 ggtcagcagt aataatcggt aacgccattt ttcttatcct catctagagc cggtgaagcc
32401 gcgtttttgc ttatatgctt tgcggtcata agtagggatg tttgtttcac gcagttttat
32461 tgcgagctgc tttctgcgtt ggaaatcgat ttcttgggtg agttcattcc aaacttttgg
32521 ataagaagtt tggaacctga acacatttaa aggcgtctta actccgtctt taactttgta
32581 aagaactgag ccattagcat tagatgcgta cacttgccag ccaatgcgga cagagtagag
32641 gcccttatca tcacggccta aaaatgacat gtagccgtca gggtgttttt tgaaattaga
32701 catgttcagc ctccttacat tcgcatgtac caacaaaggc atacgtaagc gggctaggag
32761 catctacagg tgagacgtcc ctaatattta aaggaataat ttctttgcga tatttaacta
32821 aaaccacatc accttcacgg caattgacaa ttccttctct agaagaaaaa cgtgcagatt
32881 tagaagattg ggttactctg caaaatgaaa cctcatcacc agctttgatt tttgaacggt
32941 caacaggaat catcttcttg caagtagggc agttataatc tttcattagg ctgcctccaa
33001 ccagttatta cggtcgatat agccagccaa taaaatattt atattttat ggtcatcatg
33061 attggtaaag tcattgaaag acttcccgct tagatccgtt actgaatcaa tagccaaatt
33121 tgtaatttca gcagttgtaa agtcagatcc agctacgccg tagctatcag ctacaccgtc
33181 aaaatcgaag ctcacgttta atttgaagcc gtcaatgcgg ataactgctt caccagattt
33241 ttctccagtt ttcttaacag ccagaagttc atattcagaa gcaacgactt gctcgctttc
33301 atatgagtaa ttagaaggga cgctagaatt agcagttcga tattcacaag aactcaaggc

33361 tacaagtaca gcaattgctg taactccagt taccttatgc ttgtttgaaa aggtttttac
33421 gttcataatt gatctcgcag ttttgcaaaa gcacatcgga cctggggagg ggcggtgtgc
33481 ttttttgttg tctgtgagat aaatattagg taaacctaat tattaagtca ataggtattc
33541 ctaacaaaat tagaaatacc taatttttgt gttttaatag acaaaagaaa acccacacgt
33601 ggtggtgggt agattagatg aattggatcg ctttttactt attgctcaat acttttgctt
33661 ttgcctctct tgattctttg cgatctttta atgtttttttc aagcaaagag atttctctta
33721 agtcagacca agctaagaag aagctaaaaa ttgaggtgag tccaacagaa agtattagag
33781 ctaaaagatg cttagctgaa agtaagccta ataaattaag tatatacata ctaaaaacaa
33841 gaataataaa aagcatagcc acatataaag atgacttact tcttatgtcc acagttgaag
33901 tcaacctatc tcgttctgac tgatttaaac catcaagctt taatgcatcg agcatacctt
33961 tataagccaa ataaatttga cttaaaggga gaagcagtac aaatgaaaat tgaaccagat
34021 ttattgtaat atttaactct aaatatttaa aaattattga aaatatagca aacagaccga
34081 ctaacactag tgcgataaat ctagcattat tgtagaaggg taagtagcgt ttagccatat
34141 tagtcaccaa aatttatatt ggtagtcatc caattataca attgaacttt taggccgtcg
34201 ttataaactt tattattgat tgtttcaact gaaatcttgc cgctcatttt tagattatca
34261 gccgtaacct tggtcccatc ctcaagagta ataacataat catcattatg tcgcatggat
34321 gaagctacag tatcaataac tttttgccca ctttttgagg ttttgcggtt gtatgtaagt
34381 gttaatttta actttaggtt tgcgtcatca agcccatcct ctagttttaa attttctaaa
34441 tcaactccaa aagctgactt aagcacatca accacattat cttcaatttt atagtcaatt
34501 ttcgcaggta tgcttgattc gacagtatga acaggttgaa gttctgttga cccaatgcca
34561 gatgagattg aaatagtttt agctggagtt gattcaagct tttgcttaac agtgggattt
34621 ggagcatctt ttaatattaa tgcactattc tctggcaatg ctttggctgc ttcacccaaa
34681 agccagccca aatatgactc caaggttcta gcggtaagtg atcttgattg aatgattgcg
34741 acatggttat caataacacc aaagtaaaga acactatcaa taaactcttt tcttacaacc
34801 tctgtagcgt caacgccatc ttcatcatct ggcagatcat ctgttaaata ggttttaatg
34861 ggaaattcag tagcatcatc actgtctatc tttaatacag cctgagcttt gcctgactca
34921 acaataatca actcgccaaa gaacatgctt tggtgtgaac tagcatgatt aattagaata
34981 aaatcatctt ttgttgcgga gacaaattgt tgacgattta tggctttata gtaaaaagaa
35041 tccttatcca gcaattgtgc ttttagtaat tgacctagat tggcgccttt tagaaaatct
35101 acttttttgt agtgtactgt tttgtctttt actactgttt tactcattat ttcctcctcc
35161 cgaatcgctc taaagtactg tgtagggttc acaacttatt aatctttggt gttgttaatt
35221 ttatgcccaa gctttccttc ttttaccaac tgcactactt gttcatttgt aaggactgga
35281 ataaagactt tatcaccaat gtcctttgag aggatcttca cttcttcggc tgttagcacc
35341 aaagcttcac catgtttcgc agcatcattg atgcgagcaa taatctggtt gattggtcgt
35401 tttgaattgt ccataagtct tcctgtgatt aatgcgaata aggatgttct tgtctatgct
35461 gacttggcgg cacgatatct gtaatagcgg taatactttc aacctcgtcc atttcaaaga
35521 aaaatcgctc accaccattc acagaaagca aacttaaaac cccaccattg atgccgacaa
35581 attctttaat tgtgcatctt ccatccttca agcacacctg aacaaactca ttcggcacaa
35641 gatctgcatc agggtcgcac acaacgtacc aaccattacg aattgctgga aacattgagt
35701 cgccagtgcc tttaatgcca taggctcttg gtcctgctga gtgggttgga acatatccat

35761 caccagcatt tccttcataa cccatgtcag taaaataacc atccattccc atcttggagt
35821 acgcctttac aggaacccaa cgtttagatg atggggtaaa aggtttttcg ataattgttg
35881 aaaataaaag agcttcatca ctatcactaa tgttgtattt ctttttgaat gcttcaatat
35941 ccagttgttt aaatttatct ctcgtgcttg attgaatctc tcctgtgcca gatgcaagcc
36001 aagaaggatt aacattcaaa aattttgaag cacgtaataa attttcacct tccattgttt
36061 tggattttcc agacagccaa tcactcacag aaggaggttt gactcctact gcacgagcaa
36121 gctcaacacc tttaatcttt ttaggtggca aaacttccat agcataccta agtcgttcag
36181 caagagtatt catacaacta tcctcacaat gttaggaaat cctaacataa ataaaattag
36241 gtattcctat tgatttatca taaggaatgc ctaataatta aagaaaaatt aggagcacgt
36301 tatgaatgac gcacaactta tagacaagct aggtggtgtc acagcggtag caagacttct
36361 agggattact ccgccatcag ttagtggatg gaaagctatt ccccttgata aaaaaatcag
36421 gttagcagtt attgctgaag atcttggttt aacaacacga aaagagcttt tccctgataa
36481 ctatcaagat atttggattg aactacgtcc ccagacgaca aaaagtagaa accttggatc
36541 attaaccgct taggacctta accatgagca aagtatcaaa tgaattgcct gcaagcgcta
36601 gcaataacga atcgctcata ttgcaagcgc taaatactag caaccaaaga caagtagccg
36661 agaaggtggg aatagatgca agcaccttgt cgagaatgaa aaatgacaag aaaaacaatg
36721 gattgacaga gattgaattt attagctctt tgttgacagc cattggattg aaggtggtgc
36781 cagaaagtga tgtgtattgc tcacctgaaa ttgcagaagc aacgcgagtt tatttagcac
36841 atgcattcac ttcacctgaa tacatgcgga ttttattcaa ataaaaaacc actacctgcg
36901 ggaacaggag tggttaggca ttcaaatgag gtggatcaaa tgaacacaaa caatttatca
36961 gaacaaccaa tcgaactcaa ctcaccagat tttttaatag gtgacgttgt agtacttact
37021 aaagagtgtc gaagttttaa atcaaacgat ttatttgagg ttaaaaaataa aactttgact
37081 aggttgtgga ctatcaaatc ggagaatcat ttgattctgg tttcatcaaa agaaatccgt
37141 acagcaacag tagcagagct caacgctaaa cgccgcctaa caaaagctga gcaagcatta
37201 gcggaggtgt catgaatagt caatttaagt ataaacctga gtacaaacag actcaggaaa
37261 ttcagtcctt ctttgatcca gcgttagtga ttctcaatga gctacatgat cgtaaccgta
37321 aaaatctaag agccaaaggt tatgacgaaa ataatgctgc aataacgcgt gaagaatttt
37381 cacaaactat ggcacagcgt tttcgcatta atcagtggtt agcagggcag atcgttaata
37441 gtttggctaa tgctgacttg gttcaaaaat ttggtgggta tgtaaagcct aaggtcggtg
37501 tacatgagta attttgtgcc taattccttt caagtgccta atgcatttgt tgacgaggtt
37561 ttaaataaaa tctctgatgc tgcatgcaaa atttatttag ttatttgccg taaaactcgt
37621 ggctggaata aggagatgga ttccatctct ttaactcaat ttgaagagat tacagggaag
37681 agtagaccga cagttgttaa atgccttaat gaattaatta aagttggttt agtcgtggaa
37741 caaccaagca caattcatgg aaatacattc aaattaggta acgatactag cgttggttta
37801 gtgcttaaat tccctagtaa aaatttttta ctacctgaaa tttatggcca aactagtaaa
37861 aattctttac cactgctagt taaaaatttt aactacacta gtaaaaattt tttaccgcta
37921 ctagtaaaaa tttttaacac acaaagtatc actatcaaaa acaactctca aagtaataaa
37981 aaaataaata aaaaaagaga gcctgtttct gaaaaaccta aatcagaaaa accaactgaa
38041 tttaatccac gtttagttga actaccagca tgtgtagatc cagagctgtg gaacaatttt
38101 gttgatatgc gtatcagcat caaaaaacca ctttctgaaa atgcagtaaa gctaatcctt

38161 aaaaaactta tctcgtttgg ccctttggct aaccaatcac tggaaaactc aattatcgga
38221 aattatcagg gtgtatttga gcctcgccaa aatcaaattc aggaaaaccc acaatctcat
38281 aacgttcctg aagaaccggg ttatttcact caaatgtacg ctgagagcaa ccgttcaaac
38341 gtgattgacg ttacgccagt gtcacaagat tttggaggct attaatcatg aatgaattag
38401 caccatttga aagtttttta aaagaactaa ttgcggctta cagaactaaa tacgctgttc
38461 agttcaataa gaattttcca gtagagggga aaaatgccgt tccaatgcaa atcgttgaac
38521 agcagcttgc taaagcattg gttggggtta cacctaacca acttcaaaga ggcttagcgc
38581 tattttacgc aagtacaaat acatacatgc ctaacttcgc tgaattccgt gctatgtgca
38641 tgggtgatga ttggtggagc gctgagaagg cttgggttaa ggcttgtgaa tacactcaga
38701 tctctcaaca caaaaaagtg acattgccag acggaagaga gcagaaccaa gaaattacaa
38761 ccttgaccaa atttgtttta gaccaagttt attcactaat ccaagatggt gaaatgtaca
38821 aagccaaaat ggaatttatc aaaatctatg atgaatacaa ggctgaagca caactgaagg
38881 gtaaaaccca agcttggtac caagaaccaa ttttattagc tcagaaaaat gagcaaaagg
38941 tgcataagcc tgtttcaaat gacgaagcgc aaaagcatct ccaatcattg atggaacgtt
39001 taaaaatcaa tggtcgtaaa cctgtaccag tacaaaagct taaggctaag gaaaaagagc
39061 cagaactcaa acaagagcta ggtccagatc cttttgacaa tccgcacgaa tacgcagaga
39121 tgtgccgccg tgaaggtatg ccaattccta gaaatattct taagttaatt gatggggcga
39181 atatatgaat aaattcgaga ttttagcgtg gggtttactc atttcatttt ttacagcagc
39241 tattagcggt gcggtggttt ggtggtggtt ggcgcgtaaa gagcttgatg agaaaggagc
39301 cagccatgaa agcaactaaa ttgattagag ataaaggact gcaatacgcg aaggaaatcg
39361 tagattcagc acccgataac gcaactgaat ggaacgaggg ttatgagttc caatgtggtc
39421 aaagtgtaga aatcagccca gcagatcgtg agaagtattt tgtagatttg gttgagctta
39481 aacgtctggt ggagtctttg aaaatcatca acgatttagg tggagttgag aagctaacgc
39541 ctgcattcat tacgacagat aagcatgttg gttacacgca tgttcgcatg gtgggaaatg
39601 ggagattgag ctttcttgat gatttttgcg acttcattcc agatggttcc atttcaatta
39661 agcgtgtgat gactgctatc cgcgaccacg aatcaatata cggaggcggt gaatctcatg
39721 ccaactagat ataacacagg cgagtatagc tacgatcttg aatatcacta tggagatatg
39781 tcagcaagca tggagatgct tagagcacgt ttaattgaat tgttgactcc tcatctgtct
39841 ggccgttatg tgaaatggag agaagcatat ttcacatggt ttacaaagtg cggcggggat
39901 tcggggtgga tgttttgtgt aggtccacac gaatttcata ttgatggggc gttaaggcgc
39961 tattactcag gttctattga tattacctac aaccagaaag atcgatattt cttggtgggt
40021 gagaaaaaga aagtcaaatg taaggcttgt aaggggtttg gcttcattcg agatgatggg
40081 tgggggcata tagataaatg tgaaatgtgt gatgcagaaa aaggagccag ccatgagtga
40141 gtttgagggt aaatctggaa agtgggcttg ggagattcaa aaagaacaac aagcgaaagt
40201 ggaggagctg caaaagcgtt tagatggggc attaaaagag actcaatatg ctttgcagta
40261 tgttgaagaa gacatgcgcg gcaatcatga atttctacaa atggcaatga ttcgaaccct
40321 taaagctata gagcaagtgc tcaaaggtgg tgcttgatgt catcagtcag cattgctgaa
40381 taccgcaagt tatttcccat aaagaaaaat aaaaagcgcc gttcagcaaa gcaagttgcc
40441 agacaaccaa gtgtgggtga agtggttctg gcaacgcatt taagagcatg caagattgga
40501 tttgaacagg aatataagtt ccatcctgaa cgcaaatgga gagcagattt tttaataaag

40561 ggttcaaaga ttttgattga ggtagaaggc gggatctgga gcggaggccg tcacacaaga
40621 ggtaagggct atttagggga tatggagaaa tacaactccg cagcaatgat gggttttaca
40681 gttttacggt tcagcacaga gcaagtgaaa tccggtatgg cattaaagca aattgaatta
40741 ttaattaagg gtaaatagga aggcgattat gttggttgaa aagtttgatt ttattgagtt
40801 acttcgcctt gctattgctc aaggcaaagc tgaaggtaag aaaatttcta aagatgtagt
40861 tttaggtgaa ttagcgctgt tatcgccagc tgcaaagctt tgggccactg tcttgattga
40921 aaaggttgat tttgagcgaa tcgcaataat taccccagca caaaaacaga ctgaaacttt
40981 ttacagtaag tatgacttta attttcaaac tgaacgccgt attgaagata ttccgggcaa
41041 ggttgagttt gttcgtggtg agattaaatc aggtaatttt ttcagagcgc gaaataaatt
41101 agcggtagag attcataaag aaatggtaaa gaaaaaattt acccctacta atgcccaagg
41161 tgatcttact aatctggcaa aaggtatggc tgagattatt ttgcgtggcc atgtttttgt
41221 taaggctatg tgtggagtct gccaagggtt gggtaaaatt gagacatttg gtttaaatgg
41281 ctttccaaat ggggcaaggt tttgtgaaaa atgtaatggt actggaaaac gaccatatac
41341 gttgaaagag aaaatgaata ttgctggcat tgatgcaacc aaaacagctt atataaagag
41401 ttatcagaaa tttgagctgt ttggagaatc aatcgttgct gaatgggaaa atgaaattag
41461 aacgcgtatt tctcgatcat tccgttttga acttcctgat agtcaagaaa cttacgcttg
41521 acagttgggt atacacttga gtataaagat ttctaaaatg ggcgaaatgt aaagtaatcg
41581 ccagaatgaa tttaagagct cgccaatcgg tgggcttttt tatttttaaa tatttcagta
41641 ttatcagtgt gttgctttaa gtaacactaa accttattga tcagcgcaaa tatcaaaaaa
41701 aagggggagc ttgcctacta ggcaagcttt ttaaattgat gatttaaaca caataatcca
41761 ttttaaagct caatagaaag atcaaacttc catagctttt atttgtacta atttattgaa
41821 tataatcgtt tttataattt ttaaaatttt cttaaactaa aaatggaaaa tttcttgttg
41881 caacattgtt ataataggac taccttaaga aaaatacttt ataaaaatga ggagctgctg
41941 aaatgacaca gtatctcatg tttgcggaaa atatttataa caaaattaaa gatgaggaat
42001 tgttttcaca tgactgtatt gaaaatatga acttacttat gacatgtata cgcagagaaa
42061 ttaagggaac agaatttaaa ttaaaatata attttattga ttttgttgaa ttgtttagta
42121 gaccattaga tgaatgtaaa gtaaaaatag atgttagttt gattcctcct cataattcag
42181 aaggtgagta tattttatgg ttagctggat taatcgaaaa aattacagaa ggtggaccta
42241 aaccacctcc gcctataaag aagtttattc cagagtatat gagcttgaaa tttgaattag
42301 attttttacc cttaaatgag gaaaaaattc aaaacgaagg taaagaaatt acggattact
42361 ttaattcaaa gctttataag gcaactttta agaagtaata ctatattgcc tgtgagttta
42421 gccaccgcct aagggcggtt ttttttatgg gtaagaataa tggattctac agaatacttt
42481 tggcttactc ggaaaaaaga acctaaaact aaacctaaaa gccggccatt gcctaaggcg
42541 aagcaaaaat atctcgaggc tgaggcaaca cttaaggaag agcttgagga tttggcgatt
42601 ggatttgaaa gtaagtttca gccgatccat accaaacact ggcgctttga ttttcatata
42661 gtgaaattgc gtttgctcat tgaaattgag ggtggtccct ggtctggtgg gcgtggtgga
42721 aagctgtcaa ataaagcatg gagtcttaat cgatatgatc atgctgaaga gatgggttac
42781 aaaatagagc gctttcatcc agattctatt ttgtcgggat atgtcatcaa ctggataaaa
42841 agtgaattag cgagaattga agatggagca gatcagacca tttccaccga ctgattttat
42901 tgatcaagct gaagaagagg aagcaattag actaacacca gcaccggact taaaaaaatg

42961 ggttgtggct aattacttaa ctattggggg tcctatttat aatccagatc atgatcatat
43021 tgctgagctg cttcatgata atgacgagtt tttagcattc gcgtgggcct cttctgcata
43081 taaaagcaag caagctatgg tgttaggcca gtgcgaaaaa gtcatgttca atgttggtgg
43141 ctggcgtaaa gctcgacaag agcaacagat gcgtgattgg tttggttttg tacctactta
43201 tttaataact gtcgacgctt ctttctgtga gcgtgcaaac gatacagagt tctgttactt
43261 acttgaacat gagctttacc acattggagt gatgagagac gaggacggag aaattgttta
43321 tagcgatagt tctggtcttc ctaagcacta tcttgctggt catgacgttg aagagtttat
43381 tggcgtagtt aaacgttatg gaccaagcaa aaatgttaag cgacttattg aagtcgcaaa
43441 aaatccgccg tttgtttcga atcttgatat ttcaaaatgc tgcggcaact gtgtaatcaa
43501 ttgagcctaa tggctctttt ttttgcccat tttgttatac gtagttatac gatgaggaag
43561 ttatggcgac actaaaagag cctgtgaaaa tctttatagt tcagtctctt gcttgtcgtg
43621 atacacctca agaagtggct gaactcgtaa aacaagagtt tggcgttgat atagatcgtg
43681 ttcaagttgc aacttatgac cctacaaagg ttgctggtaa gaacttaagc aaaaagtatg
43741 tcgaactatt tgaaaaaacc agagatgagt ttgataaagg cttaattgat attccaattg
43801 ctaataagta ctaccgattg aagcaatacc aaagacaact tgagaagact agaaacgtca
43861 aaacagcctt aaaaattctt gagcaagccg ctaaagacat tggtggtcaa tttactaatc
43921 gccaagaaat tacaggcaaa gacggcggac cagtccaaac agttaattca gaaattccag
43981 ttccaatgga agattactta aaagcgcgga gggaagtctt agatgagtac tgatgcggct
44041 cgggataaag ccatccggat cgaggcgcaa gaagatttat atttcttcac aaggtacatg
44101 tttaaggagc gccgtggtta taaatggatg caaaattggc accacttaga aatctgcgaa
44161 gctttaatga aagtttatcg cggagagata aagcggttaa ttattaacgt tccaccacga
44221 tattctaaaa ctgaaattgc tgtaattaat ttcatggctt ggtgttttgg taagaatcca
44281 gactgtgagt ttattcatat cagttactcg gcaatgcttg ccgcaaataa tgccttccaa
44341 atacgaactc ttgtacaaga agaggcgtat agaaaagtct ttcctgagct tacattgcgt
44401 gatgatagta aggctaaaga cttctggaga acttctcaag gcggtgtctg ctatgcgaca
44461 ggtacaggcg gtacgattac tggttttggt gcaggaaaac ttcgtaaagg ctttggtggc
44521 tgcattatta ttgatgaccc acataaagca catgaagctt catcaaaaac tattcgagaa
44581 ggggtaattg attggtttca gaacacactc gaatcgcgta ctaactcgcc agatacgccg
44641 atcattgtga ttatgcagcg acttcatgaa gatgatttag ctggatggtt gctaggtgat
44701 agaaaagacg gcgttcctgt agctggtggt aacggtgaag tgtgggagca tctatgtctt
44761 tcagctattc aggaagacgg atccgcactg tggccagcaa aacacaatat ccaaaaattg
44821 aggctaatgg agcaagcagc accatatgta tttgccgggc agtaccgaca aatgccatca
44881 ccgccagcag gcggtttttt taagcccgac aatattcaaa ttgttgatgc tttgcctgcg
44941 gatgtagtga aacaagttag ggcttgggat tttggggcta ccgaaaatga gggcgacttt
45001 acagtaggtg tgcgagaagc tctaggcgca gatggtttta cttacattgt cgatgtaact
45061 agaggacagc ttggacctga caatgtgaat aagcgcttag aacaaacagc aaaaatagat
45121 gggaaaaaag tttctgtgcg tctaccacaa gatcccggtc aagctggtaa atcacaagct
45181 agttcatttg tgaagcttct tgcgggttat agcgtgatag ctaagccaat ttcaggtgac
45241 aagcttacac gtgcacaacc atttgcggcc caagttaacg taggaaatgt acgaatgctc
45301 aaaggtgaat ggaataagga ttttattgat gagcttcgtc attttcctaa tggcacacat

45361 gacgaccaag tggatgcagc ttcagatgcg tttaatgaat tacatgaagg ttttgaagcc

45421 ttctttgctg atatgggatt tgctcgatga gtgatgtaac ttttcaacat gctgaatatg

45481 ttaagaactt gccatactgg caaaaacttg atgatgtttg tgaaggtgaa gatgcagtta

45541 aggctaaagg tgaaaaatat ttgccgatgc caaatgcaca tgataaatca cctgcaaata

45601 aaagcgctta tgaggcttat cttacccgtg cagtctttta tgaagtaaca gggactacat

45661 taaatagttt agttggtgca gcttttgcaa ccgatccaag ttttaaattt cctccggaac

45721 ttgctcattt agaacgtaat gcaaatggtg ctggtttaag tacttatcaa ttggctcaaa

45781 atggaattcg ccatttattg aagcattatc gttgtgcttt atatgtagat tatcctgatg

45841 tgccgccagc tcgtaatcta gcggaattta aagcacaaaa agcctatccg atgattcatt

45901 tactaaatgc ccttgatgta gtgaattggg attcagtaat gatcgataac cagaaaaagc

45961 tttgcttagt ggttatacgt gaatttaagt ctgagcgcgg tgctgatgga tttagtaaaa

46021 ccgaacaaga gcaatatcgt gtacttcgtt tagagcaaga gggaaatggg gaatatattt

46081 attccgttca ggtgtacaca aagggtgaaa agggtaactg ggttggcgga gagaagaagt

46141 ttccaacaga ttacaacggg aatttctgga cctatatacc ttttacattt gtaggtgcaa

46201 ttgataattc agaagagatt aaaaagccac cattacttcc tttggctaat ctcaatttag

46261 cccattacag agacagtgcg gactttcaag agtccgtttt ttatatgggg caacctcaat

46321 attatgcgaa gggtgttaat tgggagtggt atgaccaagc caagaaacgt ggcatctaca

46381 ttggagcgaa agtacttttg cctttacctg aaaatggtgg tttaggaatt gtacaagccg

46441 accctaatac tcttgcccgg gaagcgatga aagataagtg ggaaaaaatg aaggagatgg

46501 gggcgcgttt aattgagaag ggctcgggaa gtaaaaagac cgctaccgaa gcgaatagtg

46561 atgacgccgt tcagcattca gttctttcgc tctgtgtcgt taatatgaat gaagccttgt

46621 cagcagcatt acgatgggct gctaagtttg taacgcctaa tgtggatgtt ctaactaaag

46681 atgatttgat gttcgaaatc agtcaagaat ttaacaaaca gggttattta gctgagttag

46741 ctcgacagtt atttgaagca gctctacaag gccgatcttc atttaaatca tggtgggaat

46801 acaaccaaac aggtatgttc cctaaacaaa aatatgaaga agagcttcag aatgttgaag

46861 cagagcaaga tgggacttta aatcaaaagg tagagtgaga tggcaacaga tatcaaaaaa

46921 ctatttgaag tactcactca gcaccaggcc tatctttatc gtgcttcatc aaaaaacggta

46981 aatgagttat tggctttatt caatgatgat acgagcaaga tgctatctaa gcttcgggat

47041 ttattggatg agcttaatga gtcggagaaa gttgctttag ctggtggtaa atatacaact

47101 tcaaatttaa gggaaattag ggatttgatt gcccaatggt ttgccagtgt taatttagca

47161 ttacctgaag cttttgccgt ttctgctacg gcgctggctg tttatgaggc caattacgta

47221 gctaagctct atggagcaaa aattaataag cctgatgggg aaaaactatt cttatccgct

47281 aaaaaagttc cgttggcagg tggcgctctt gtcgatgatc tgctttcaag aattgctgaa

47341 agtgcccgtc aaaaggttga gtatgcaatt cgagatggta ttaattcagg caaaactaac

47401 caagaaattg ttcagcgtat tcgtggtacc aaacggctta actatgaaga tgggatctta

47461 aatggtacca aaactgatat tgagcgaacg gtaagaactg tgcgaagtca tgtagctaat

47521 caagcctatc taaatagctt caaccaaatt ggctttgaat atgtccgatt tgttagcgtt

47581 ttagatggac gaacttctaa gctttgcgct tcattagatg gttcagtgtg ggaaataaat

47641 gatccggcaa agcgagtgcc gccgttacat cctaactgtc gcagtatctt ggttccggtc

47701 gagaaggacg gtcaacttgt tggcgaacgg ccatttgtaa tggacgaacg tagagttaaa

47761 gacatcccca aagaagagcg aagccagtta ataggacagt tagatgcaaa caccacattc
47821 aaagagttct ttaagaaaac agatgatttc tttcaaaggg agtggctagg gccaaagcgc
47881 tttaagctct ataaagatgg gaaatttgat tttgataagt tctttgatcc tgaaggccgt
47941 ttctatagct tagatgattt gagaaagttg gatgaaaaag cttttaaaaa gttgggtctg
48001 taatttttct tatgttatat tttttaaaac atcagaattt atacaatatg aaaacaatag
48061 cttttgtatg tctaacccta atttccatca cttgtttagc tgaaccaagt caaaaatatc
48121 ttaaagaata tgatcgattg tctgaagctt tggagtcagc aatggcaaat gcatattctt
48181 ttgatcctgc aactggtcaa gtaaaacagg ctactcaagg tttagaagct aaaaataatt
48241 tatgtagagc tgcccaggcg aaactaaacc tcaccacgtt tttaaaagac aatttagagg
48301 aatctaaaga gctttataaa tctattgatg gtgcagagac tctagataaa aattatctta
48361 gtggacaaca gcaggaacaa caaaatctcg tttcaaattt gaaaaaagac cttgttggaa
48421 ctggatttaa ctgtgagtaa ttattgccga ttacaggtaa ttctaaactc acttaagaca
48481 caattttcac ctatataagc gcccaaatgg cgcttttgtc atttatggag tttggcttat
48541 gagtgaatca aaagttagac atttggtact taaaagagtt tcagataaat cttctcatct
48601 tgctctttgt gacgaggaaa caggtattcc attagctgga ttaaccgctg taaaaatgaa
48661 ttgtagtgtt tttgagggtc cagcgactat cacggcaaca tttgatgtag gtggtcctca
48721 aggcatccgc ttagttggtg atgaacctag acaaaaggtt tggagtgcaa aggaaacgta
48781 gcgaaaggca ctacaaatgc ctgaaaagca aatcaatatg tcagatgctc aatatattct
48841 gagcacaaaa tgaattctgg tgccatttct tcaaattaag gtttcaagcc atggcaattt
48901 atggttttac ttttgaaaga ttaaaagcaa ttgcactcat caaatagaac ttaattttta
48961 accatagcac cttcgggtgc ttttttgcg agaagaaaat gccaagccct attatccaat
49021 atttccaata tgaacattta cctgaacatt tgcagcaagt tagtaagcca attggtgatt
49081 tagctcggca aatggatgag caacttcctg acgggcctga aaaatccaca ggattaagaa
49141 agctacttga agcaaaagat gcatttgtac gccaagcttt aagtaaataa tcatttatag
49201 aaatgaagcg tcctaaaggg cgcttttta ttgcctgccg aaagcggatg ctaacggcga
49261 atccgggcgg atgcccattt tgtatatata ggttggatga ccaatgaaac ttaaaacagt
49321 aacaatcgac ggtaaagttt atgcggaagt agacggtgat aagccgatct atattcatga
49381 tgacggcaaa gaaatgccac atgatgcacc acactcggta gcaacaattg cacgcttaaa
49441 caatgaagct aaaacacatc gtgaagccaa agaagcagcc gaaaaagcat taaaagcttt
49501 tgaaggaatt gaagacccag cggcagctaa aaaggcatta caaacaatcc aaaatctcga
49561 tgataaaaag ctggtggatg ccggtgaagt tgagaaagtt aaagctgaag ctatcaaagc
49621 agttgaggaa aaatatgccc cgattgttgc gcaacgtgat gctctagaag cctctttaca
49681 taaagaactt atcggcggtg gttttgctcg ttctaagtac attcaagaca acattgcagt
49741 acctgtggac atggttcagg caacctttgg tcatcacttc aaaatcgaag aaggcaaggt
49801 ggttgcatat gatccgaacg gcgaaaagat ttattcacgt gtccgcccgg gtgaacttgc
49861 aaatgttgat gaagctttag agtcattggt tggtggatac cagcataaag acttaattct
49921 taaaggtggt aaaggaactg gtggcggttt tcaaggtggg ggcaaaggtg gagcacctac
49981 tggaatgaaa cgcagtgaaa tgtctgtttc tcagaaagca gattacatca aagaacatgg
50041 caatgatgcc ttcctaaaac taccgaacta atcattaaat atttggagat aagtagttat
50101 gactacgaca gttaattcag acatgatcat ctataatcaa ttggctcaaa ctgcttattt

50161 agagcgtttg caagataatt tgaatgtatt taaccaagcc tctaatggtg caattgttta

50221 tcgcaatgag atcattgaag gtgatttcaa taaagaagca ttctacaaag tgggcggtag

50281 catcaaacat cgtgatgtga attcaaccgc caaagtagtt ccagagaaaa ttggttctgg

50341 tgagtctgta ggcgtaaaag tcccatataa atatggtcct tatgcatcaa ctgaagaggc

50401 atttaagcgc cgtgctcgta caccagaaga atttgctatg gttgttggtt acgatcttgc

50461 agatgcattg gttgcaggcc gattagagta cagtttagct tctttaaaag ctgctatttc

50521 tagcaatcca gacatggttg caaaaggtag tatcgttgtt gatggccgca aagcattgac

50581 tcgtggtatg cgaaagtttg gtgataagtt tggccgcatt ggcttatggg tgatgaactc

50641 agatacatat ttcgatattg tcgatgatgc aatcactaag caaatttatg gtgaatctga

50701 aatcgttatc tacggtggtt taccgggaac cttaggaaag ccggtcttgg tgacggacgc

50761 tgtaggtgat aacgatgctt ttggtttgca gtatggcgct gtcactgtaa ctgaatcaca

50821 agtaccgggc ttccgagctt atgacatcaa tgatgaagaa aacttagcaa tcggtatgcg

50881 tgctgaaggt gcatttaact tagatattct tggttatagt tgggatacaa

//

Table S2: Oligonucleotides used in this study

Primers	Oligonucleotide sequence (5'→3')	Use
Phage1_F	GGTGGTGGGTAGATTAGATG	Confirmation of Phage region 1
Phage1_R	CATCAAGCTCTTTACGCG	
Phage2_F	GAAACCTTTGGCAGCTTTAC	Confirmation of Phage region 2
Phage2_R	CCACCACATTCAAGTGTTAG	
Phage3_F	GTAAAATGGCACAAGAATCCC	Confirmation of Phage region 3
Phage3_R	GCTGGTCTGAGGATTTAGC	
